# Safety evaluation of FM101, an A3 adenosine receptor modulator, in rat, for developing as therapeutics of glaucoma and hepatitis

**DOI:** 10.17179/excli2019-2058

**Published:** 2020-02-12

**Authors:** Chong-Woo Park, Chung-Tack Han, Yasue Sakaguchi, Jiyoun Lee, Hwa-young Youn

**Affiliations:** 1 R&D Center, Futuremedicine Co., Ltd., Seongnam, Republic of Korea; 2 Laboratory of Veterinary Internal Medicine, College of Veterinary Medicine, Seoul National University, Seoul, Republic of Korea; 3 Biotoxtech Co., Ltd., Cheongju, Republic of Korea; 4 Ina Research Inc., Ina, Japan

**Keywords:** adenosine, A3AR modulator, acute oral toxicity, 28-day subchronic toxicity

## Abstract

Adenosine is a critical regulator of inflammation and fibrosis, it affects endogenous cell signaling via binding to the A_3_ adenosine receptor. FM101 is a potent, highly selective A_3_ adenosine receptor modulator that has been developed as a treatment for glaucoma and hepatitis. We determined that FM101 is a biased ligand with functional activities both as a G protein agonist and a β-arrestin antagonist. The safety of FM101 was evaluated by administering an acute dose in rats, the results indicated that the approximate lethal dose was greater than 2000 mg/kg. In a subchronic toxicity study, FM101 was administered orally once per day to rats at doses of 250, 500, and 1000 mg/kg/day over a period of 28 days. Abnormal posture, irregular respiration, decreased movement, and ear flushing were observed during the early phase of dosing, and loose stools were observed sporadically among the animals that received 500 and 1000 mg/kg/day. Body weight and food consumption were decreased in one male and one female rat in the 1000 mg/kg/day group during the first 2 weeks of observation. However, there were no test substance-related changes or adverse effects observed during our ophthalmological, clinical chemistry, urine, organ weight, and histopathological analysis. These findings indicate that no observed adverse effect level of FM101 was 1000 mg/kg/day in male and female rats.

## Introduction

Adenosine has a nucleotide-based structure and it regulates regulating endogenous cell signaling via binding to adenosine receptor (AR) subtypes A_1_, A_2A_, A_2B_, and A_3_. Among ARs, A_3_AR is a well-known G protein-coupled receptor (GPCR) that is expressed abundantly on inflammatory tissues, and its selective compounds are effective for the treatment of inflammation or fibrosis (Cronstein and Sitkovsky, 2017[5]; Borea et al., 2018[3]; Lee et al., 2013[14]; Yu et al., 2019[29]). Adenosine agonists or antagonists, when used in pharmacological studies, have been shown to be effective for lowering intraocular pressure (IOP) depending on the AR subtype (Jacobson and Civan, 2016[9]; Lu et al., 2017[17]; Li et al., 2018[16]). Among the various adenosine receptors, the A_3_AR could be a promising target to reduce elevated IOP since IOP reduction has been achieved through selective A_3_AR agonists and antagonists demonstrated in various animals (Yang et al., 2005[28]; Okamura et al., 2004[18]). The most promising A_3_AR full agonists, such as IB-MECA and Cl-IB-MECA, have shown a good safety profile in patients during clinical trials and nonclinical safety studies (Stemmer et al., 2013[23]; Jacobson et al., 2019[10]).

FM101 is a chemically synthesized novel A_3_ adenosine receptor modulator. It is described as a truncated analog of D- or L-4′-thionucleoside (Jeong et al., 2008[11]), and has the following full nomenclature: [(2R,3R,4S)-2-[2-chloro-6-(3-chlorobenzylamino)-9H purine-9-yl]-tetrahydrothiophene-3,4-diol]. FM101 was synthesized based on the D-4'-thioadenosine derivatives without a 4'-hydroxymethyl group; its synthesis begins with D-mannose and has key steps consisting of cyclization to the 4-thiosugar and a one-step conversion of the diol to the acetate. This novel N6-substituted analog binds to the human A_3_AR with high potency, selectivity, and binding affinity. It is being developed as a therapeutic for high IOP in cases of glaucoma and to decrease for inflammation and fibrosis in non-alcoholic steatohepatitis and kidney disease (Lee et al., 2013[14]; Shon et al., 2019[22]; Lee et al., 2017[15]). 

There have been several investigations evaluating the efficacy or tolerability of candidates targeting A_3_AR in mice that overexpress or lack these receptors (Black et al., 2002[2]; Harrison et al., 2002[8]; Xiao et al., 2019[27]). However, there is very few published studies reporting the non-clinical toxicity of agents that are highly selective for the A_3_AR and/or have biased functional activity on the A_3_AR. Thus, this study aimed to assess the safety profile of FM101 in nonclinical toxicity studies. 

## Materials and Methods

### FM101: production and characteristics

FM101 was supplied by Futuremedicine Co., Ltd. (Republic of Korea) as an off-white powder with a purity >99.0 %. It was stored at 4 °C in a sealed container with silica gel.

### cAMP accumulation and β-arrestin translocation assays

A cAMP accumulation assay was conducted using Euroscreen FAST in accordance with the service supplier's instructions. CHO-K1 cells expressing human A_3_AR were mixed with forskolin and increasing concentrations of FM101. After incubating the cells with lysis buffer, cAMP concentrations were estimated using a homogeneous time-resolved fluorescence (HTRF) assay kit (Perkin Elmer, USA).

The translocation of β-arrestin was evaluated using a PathHunter™ β-arrestin assay kit (Fremont, USA) from DiscoverX according to previously described procedures (Alnouri et al., 2015[1]; Olson and Eglen, 2007[19]). To evaluate the antagonist function of FM101, CHO-K1 cells expressing human A_3_AR were pre-incubated at ten concentrations ranging from 0.05 nM to 1 μM with a three-fold increase of concentration of FM101, followed by treatment with 0.06 μM of 2‐Cl‐IB‐MECA as a reference agonist. After incubation with the assay buffer, a chemiluminescent the assay signal was generated using a commercial PathHunter Detection reagent cocktail.

### Experimental animals and test system

For the acute toxicity study, male and female Sprague-Dawley (SD) rats (Crl:CD SPF) rats were purchased from Orient Bio Inc (Seungnam, Republic of Korea). For the subchronic exposure study over 28 days, male and female SD rats were acquired from Charles River Laboratories Inc. (Japan). Rodent pellet feed (Envigo RMS, Inc., U.S.A. or Oriental Yeast Co., Ltd. Japan) and sterile tap water were provided *ad libitum* during the experiment. Rats were housed in stainless steel cages at appropriate temperature (21-25 °C) and relative humidity (40-70 %). Animal housing facilities maintained under an automatic 12-h light/dark light cycle. Animals were allowed to acclimatize to the housing conditions at least 1 week prior to the experiments. Animal husbandry procedures followed the recommendations of the Association for Assessment and Accreditation of Laboratory Animal Care International. All acute and subacute studies were approved by the IACUC of Biotoxtech Co., Ltd and INA Research Inc., respectively (IACUC Accredited No. 160580 for acute toxicity; Accredited No. 001107 for subacute toxicity).

### Acute oral dose toxicity study

The acute single oral dose toxicity of FM101 was investigated by Biotoxtech Co. Ltd., Republic of Korea, in accordance with good laboratory procedures (GLP). Five male and five female rats were dosed orally with FM101 in a 0.5 % methylcellulose (MC) solution at a dose of 2,000 mg/kg with a dose volume of 10 mL/kg. A group of control animals was dosed with the MC solution (vehicle) only. All animals were observed at least once at 0.5, 1, 2, 4, and 6 h after treatment and once daily thereafter for 14 days. Body weights were recorded on the day of treatment (Day 0) and again on Days 1, 3, 7, and 14. After the observation period, the rats were euthanized and complete gross postmortem evaluations were conducted.

### Subchronic oral dose toxicity study

Subchronic toxicity was investigated at Ina Research Inc., Japan, in accordance with the GLP standards for non-clinical safety studies. During a 4-week repeated dose toxicity study, FM101 in 0.5 % MC solution (vehicle) was administered via gastric intubation once daily at a dose volume of 10 mL/kg. The rats were divided into four groups containing ten male and ten female rats each; three groups received 250, 500, and 1000 mg/kg/day as the low-, mid-, and high-dose groups, respectively; the control group, comprising ten male and ten female rats received vehicle only. Six male and six female rats in the control and high-dose groups were allocated for recovery studies with the post-exposure effects assessed over a 2-week period after dosing ceased. All animals were observed twice a day for clinical signs and mortality. Body weights were recorded on the first dosing day (Day 1) and once a week thereafter during the study period. Total food consumption was recorded, and mean daily consumption was calculated. Ophthalmological examination was conducted in both eyes prior to dosing, during week 4 of dosing, and during week 2 of the recovery period after topical application of mydriatic agents on the eyes. The anterior portions of the eyes, including the optic media and fundi, were observed and evaluated using a slit lamp or a binocular indirect ophthalmoscope. Blood, serum, and urine samples were collected and analyzed during the week 4 of dosing and week 2 of recovery.

At the end of the experimental period, the animals were euthanized under isoflurane anesthesia. Absolute organ weights were recorded and organ to terminal body weight ratios (relative organ weights) were calculated based on the body weight at the time of necropsy. For histopathological examination, the obtained organs and tissues were preserved in 10 % neutral buffered formalin. The testes and eyes with the optic nerves were fixed in a formalin-sucrose-acetic acid solution and 1 % formaldehyde-2.5 % glutaraldehyde in phosphate buffer, followed by preservation in neutral buffered formalin. After these steps, all organs were embedded in paraffin and stained with hematoxylin and eosin for microscopy. Histopathological evaluation was conducted in fixed organs from the control and high-dose groups and in tissues with gross lesions from all the groups.

### Statistical analysis

Results were statistically analyzed using SAS software (version 9.3, SAS Institute Inc., U.S.A.). In the acute toxicity study, body weights were analyzed using a folded-F test. Two-tailed Student's t-test was used on homogeneous data. In the subchronic toxicity study, body weight, food consumption, organ weights, and hematology and clinical chemistry parameters were analyzed using Bartlett's test. One-way analysis of variance (ANOVA) was used for homogeneous data and significant values were examined using Dunnett's test for multiple comparisons. Kruskal-Wallis test was used to analyze heterogeneous data, and significant results were examined using Steel's test for multiple comparisons. Data obtained during and after the recovery period were analyzed for equality of variance using Student's t-test. All differences were regarded as significant at p < 0.05 or p < 0.01.

## Results

### In vitro functional profile of FM101

**Figure 1:**
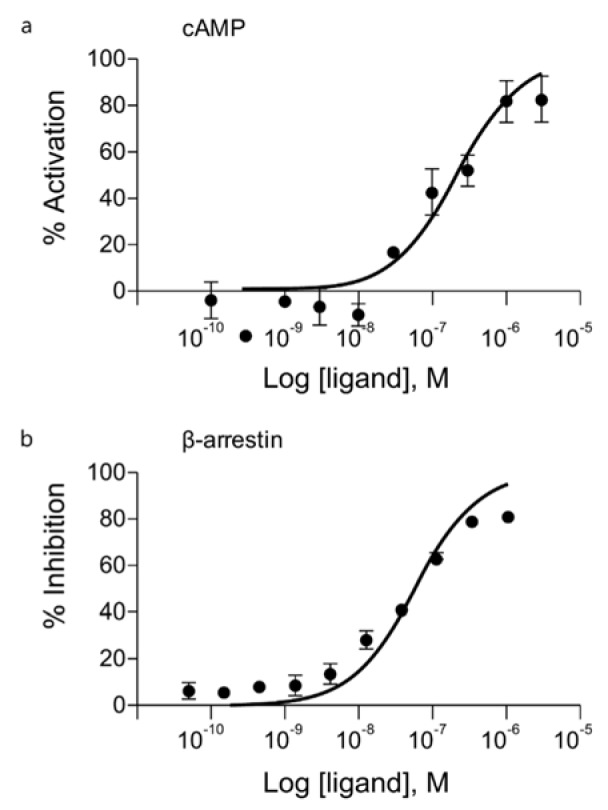
Concentration-response curves of FM101 in cAMP and β-arrestin signaling pathways. (a) Agonist functions of FM101 as measured by the accumulation of cAMP. (b) Antagonist function of FM101 as measured by the translocation of β-arrestin. The results are expressed as mean ± standard error from independent experiments. Data were from at least 3 separate experiments providing similar results in duplicate.

FM101 was identified through structure based optimization of compounds. It was determined that FM101 selectively binds to human A_3_AR with a Ki value of 1.44 nM (Jeong et al., 2008[11]). We confirmed the functional activity via the cAMP HTRF assay and β-arrestin translocation assays on the G protein or β-arrestin dependent signals, respectively. FM101 inhibited forskolin-stimulated cAMP in CHO cells overexpressing human A_3_AR in a concentration-dependent manner (Figure 1(Fig. 1)). Potent agonism of FM101, as determined by cAMP inhibition through G_α_ protein-dependent signaling, was observed, with an EC_50_ of 104 nM. Translocation of β-arrestin was measured using a β-galactosidase complementation assay. The results indicated that FM101 was a potent antagonist of β-arrestin dependent signaling, with an IC_50_ of 44 nM (Figure 1(Fig. 1)).

### Acute oral dose toxicity study

After a single dose of 2000 mg/kg FM101, all animals survived for 2 weeks until scheduled euthanasia. Stool that had the same hue as FM101 was observed 1 day after dosing in all the rats. Fecal volume in one male and three female rats was lower than other rats 1 day after dosing. Abnormal clinical signs observed on Day 1 returned to normal the day after dosing and did not reoccur afterward. 

The mean body weights among the female rats on Days 1 and 3 after dosing (142.6 g and 160.0 g, respectively) were significantly lower than the body weights in the control group (6.9 % and 3.8 % reduction, respectively; p < 0.05). However, the body weight had recovered by Day 14 (Table 1(Tab. 1)). No grossly visible abnormalities were observed during the postmortem examination.

### Subchronic oral dose toxicity study

#### Clinical signs 

No morbidity or mortality was observed in any dosing group during the treatment or recovery periods. Prone posture, decreased movement, and irregular respiration were observed in several animals in all the treated groups on Day 1. However, these signs disappeared prior to dosing on Day 2 or Day 3. Ear flushing was observed in several animals in all the groups on Days 2 and 3. In the 1000 mg/kg/day dosing group, one male and one female rat exhibited soiled fur, scant feces, piloerection, and/or irregular respiration during week 1; both animals recovered by Day 10. Yellowish-brown or loose stools were occasionally observed in the 500 and 1000 mg/kg/day groups during the treatment period. Crust formation on the dorsocervical skin was observed during the last week of dosing in several animals in all the treatment groups, including the control; however this symptom disappeared during the recovery period.

#### Body weight and food consumption

**Figure 2:**
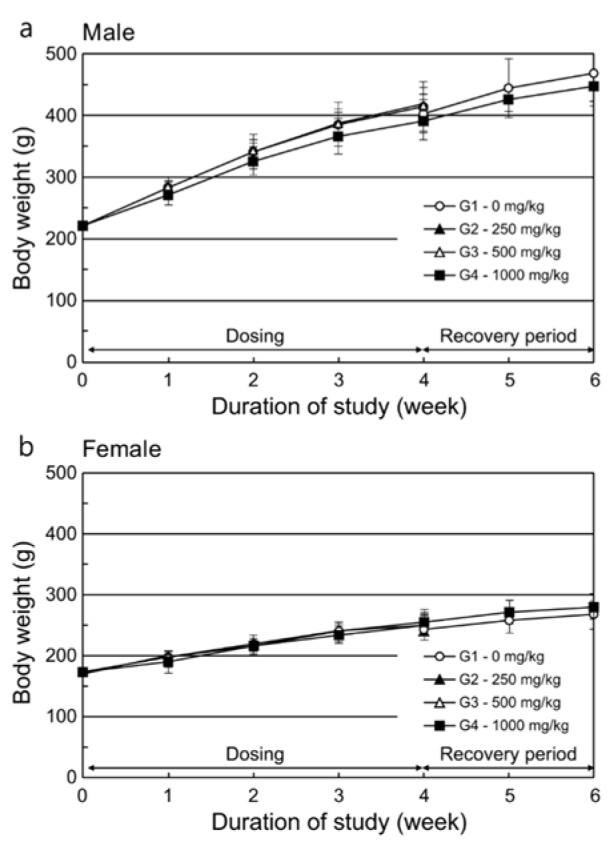
Mean body weight in (a) male and (b) female rats during the main experimental and recovery periods of the subchronic toxicity study.

During the dosing period, there was no statistically significant difference in mean body weight among the control and treated groups (Figure 2(Fig. 2)). Individually, reduced body weight gain and reduced food consumption were observed in one male and one female animal that received 1000 mg/kg/day during week 1 of dosing. Their weights were significantly lower than the mean weight of the control group or other animals in the same group. When the body weights of these animals were compared between week 1 and the first day of dosing, the male animal gained 6 g (compared to a mean body weight gain of 61 g for the control group) and the female animal lost 42 g. However, this reduction recovered during week 2 of dosing (Table 2(Tab. 2)). Therefore, these values were considered outliers and were not included in the calculation of average body weight or body weight gain in their respective groups. All other changes in body weight in the animals of the 1000 mg/kg/day group were not significant except for the body weight reduction observed in the two previously mentioned animals compared to the control group.

#### Ophthalmology 

White dots or spots in the lens (anterior cortex or nucleus), opaque the cornea, and retinal folds or zonal hyper-reflection in the fundi were sporadically observed in both the controls and the experimental groups during week 4 of dosing and week 2 of recovery. These observations were considered spontaneous or incidental as there was a lack of dose-dependency, and the rate of incidence did not differ significantly between the control and experimental groups. 

#### Hematology

During week 4, the mean corpuscular volume and mean corpuscular hemoglobin increased significantly in male rats receiving 500 mg/kg/day (Table 3(Tab. 3)). The red blood cell count and prothrombin time decreased and absolute or relative neutrophil count increased in the female rats in all the treatment groups. The absolute monocyte count significantly increased in the female rats in the 1000 mg/kg/day dosing group (p < 0.05). The hemoglobin levels, basophil ratio, and number of monocytes increased in the female rats in the high-dose recovery group compared to the controls (p < 0.05). In males, only the basophil ratio significantly increased (p < 0.01). However, these changes were within the normal variation and there were no relationships between sexes, which suggests that these changes were not FM101.

#### Clinical chemistry and urine analysis

At week 4 after dosing, the level of triglycerides in male rats was lower in the 500 and 1000 mg/kg/day groups, whereas the total cholesterol levels in the females were significantly higher in the 500 and 1000 mg/kg/day groups (Table 3(Tab. 3)). However, these changes were within normal variation and there was no relationship between sexes, which suggests that these changes were not related to FM101. Total bilirubin levels were higher in the male animals in the 1000 mg/kg/day group. Furthermore, significant increases in total protein were observed in the female rats of all dosed groups. However, these significant changes were slight and considered to be incidental, spontaneous, or within the range of historical data from the test facilities. Urine analyses during week 4 of dosing or week 2 of recovery indicated occasional increases in protein, the presence of occult blood, and the presence of ketone bodies in the treated rats. These results were similarly considered to be incidental as the responses were noted in the historical data of Ina Research Inc.

#### Organ weights

At the end of the dosing and recovery periods, several parameters were significantly different compared to the control group (Table 4(Tab. 4)). For instance, in the 1000 mg/kg/day group, absolute heart weights were lower in males, whereas the relative heart weights were higher in females. Relative liver weights were higher in females receiving 250 mg/kg/day, whereas the absolute and relative liver weights were lower in males receiving 1000 mg/kg/day. In the recovery groups, the absolute and relative weights of the thymus as well as relative weights of the lungs, kidneys, and testis were greater in male rats receiving 1000 mg/kg/day compared to the control group.

Although the above differences were significant, they were considered incidental or spontaneous because they were within the range of historical data from the test facility and there was a lack of supportive findings in clinical pathology, histopathology, and other examinations.

#### Gross findings and histopathology

Dorsocervical skin crusts could still be observed in one or two rats per group at doses of 250 or 1000 mg/kg/day at the time of necropsy after 4 weeks' dosing. In one male rat in the control group, a nodule on the spleen was observed. Among the recovery group animals, a dark reddish focus on the mucosal layer in the glandular stomach was observed in one female rat in the 1000 mg/kg/day dose group. According to the histopathological evaluation, inflammatory cell infiltration and/or pustules found in the dermis or subcutis were related to the crust formation that was macroscopically observed (Table 5(Tab. 5)). Furthermore, the focal lymphoid hyperplasia found on the spleen corresponded with the macroscopically observed nodule in the control group. Other findings were not considered test substance-related because their incidence rates did not significantly differ from those of the control group. Further, they lacked dose-dependency, had a low degree of differences between the control and high dose groups, or had other indications of spontaneity.

For more results see the Supplementary data.

## Discussion

Our findings demonstrate that FM101 is a biased modulator of A_3_AR with functional agonism toward G protein activity and antagonism toward β-arrestin recruitment. These results also suggest that FM101 is safe and well-tolerated upon single and multiple dosing in rats. Based on the results of the safety assessment in this study, a Phase I clinical trial of FM101 administered orally to healthy volunteers has been initiated (Lee et al., 2018[13]).

As a member of the GPCR family, A_3_AR is highly expressed on the cell membrane. Adenosine, which is released in the extracellular space, binds to adenosine receptors on the target cell surface and causes the dissociation of the G-protein subunits G_α_, G_β_, and G_γ_ (Chen et al., 2013[4]). The G protein regulates the activities of secondary messengers and plays an important role in transferring extracellular signals to the cytosol. The intracellular signaling pathways activated by GPCRs, including A_3_AR are regulated by β-arrestin as well as the G protein subunits (Reiter et al., 2012[20]). As a result, the concept of the functional selectivity of GPCR has been established; this concept suggests that, depending on their signaling pathway, different ligands of GPCR exert different efficacies or safety profiles when binding to the same target receptor (Urban et al., 2007[25]; Tan et al., 2018[24]). FM101 has been functionally characterized by its ability to affect the G protein and β-arrestin pathways. It displays biased functional activity: its G protein agonism inhibits the accumulation of cAMP (EC_50_ = 104 nM, E_max_ = 82 %) and prevents the recruitment of β-arrestin (IC_50_ = 44 nM, E_max_ = 81 %). A_3_AR antagonists against cAMP signaling and partial agonists for β-arrestin recruitment have been identified as functionally biased ligands at the A_3_AR (Gao and Jacobson, 2008[7]). However, a functionally biased ligand that is an A_3_AR agonist with regard to cAMP-dependent signaling and an antagonist for β-arrestin-dependent signaling has not yet been reported. Therefore, this study provides, to our knowledge, the first safety profile possibility for a biased agonist of A_3_AR.

The amino acid sequence of A_3_AR demonstrates substantial variation among mammalian species (Alnouri et al., 2015[1]); for instance, there is only approximately 71-74 % sequence homology between humans and rodents (Klotz, 2010[12]) and about 90 % homology between rats and mice (Alnouri et al., 2015[1]). Due to these species-dependent differences, many potential A_3_AR agonists/antagonists have shown lower potency or activity in rodents despite being highly potent in humans (Alnouri et al., 2015[1]; Klotz, 2010[12]). Therefore, rodent models may not be suitable for such studies. In this study, FM101 was found to be a species-independent and selective ligand for both human and rodent A_3_AR subtypes. This allows us to use rodents as a model for the evaluation of the toxicological profile of FM101.

In a previous experiment to develop new chemical entities targeting A_3_AR, a full A_3_AR agonist was found to be safe and well-tolerated in humans and animals. CF101, a selective A_3_AR full agonist that is currently under development for rheumatoid arthritis and psoriasis (Fishman and Cohen, 2016[6]), was well-tolerated in nonclinical studies and Phase I clinical trials with healthy subjects (van Troostenburg et al., 2004[26]). CF101-related clinical observations were made after a single dose of 10 mg in healthy volunteers with symptoms of flushing, tachycardia, nausea, and vomiting observed. Headache, drowsiness, flushes, and dizziness were also observed among healthy volunteers receiving multiple doses of 5 mg. These observations diminished over time in these studies.

In our acute toxicity study, FM101 was well-tolerated, suggests that the approximate lethal dose of FM101 is likely much higher than 2000 mg/kg. Furthermore, over the 4 weeks dosing period during the subchronic toxicity study, clinical signs included flushing, irregular respiration, or prone posture. However, these symptoms diminished over time within a week, which was similar to the observations with CF101, despite the much higher dose levels. As such, while these signs are considered test substance-related, they are not toxicologically significant. Moreover, these signs disappeared within a week during the early stages of the dosing period and did not affect general health or cause changes in clinical pathology or histopathology in the 250 or 500 mg/kg/day groups. Although changes in fecal characteristics were observed in the 500 and 1000 mg/kg groups, these findings also lack toxicological significance as they were only observed during the first two weeks of dosing and did not affect the digestive system according to pathological or histopathological findings. Overall, FM101 showed similar clinical outcomes with a potentially superior safety margin compared to the full agonists, as these signs were observed only occasionally or for a short duration. At 1000 mg/kg/day, we observed deteriorated physical conditions, decreased body weight, and reduced food consumption during week 1 in one female rat. We also observed slower weight gain and decreased food consumption in one male rat. No changes suggestive of deteriorating physical condition were noted following clinical or histopathological examination. These findings were considered to be the effects of treatment with the test article, but were limited to only two animals, both of which recovered within 2 weeks of the dosing period. As such, we do not consider these findings to be toxicologically significant. Additionally, we will evaluate and assess the correlation of these signs with toxicological significance in future multiple-dosing studies over long term.

A_3_AR is densely distributed in the human lung and liver, but less so in other tissues (Salvatore et al., 1993[21]). In rats, it is highly expressed in the testes (Zhou et al., 1992[30]). We evaluated the effects of FM101 on all of these organs. We observed a significant increase in the liver weight among females receiving 250 mg/kg/day and lower absolute and relative liver weights among males receiving 1000 mg/kg/day at the termination of the dosing period. As with previous findings, these changes are considered incidental due to a lack of dose-dependency or supporting changes in clinical pathology or histopathology. Additionally, no test article-related findings were observed in the testes or lungs. According to the ophthalmological evaluation, white dots or spots in the lens, opacity of the cornea and retinal folds, or zonal hyper-reflection in the fundi were sporadically observed in the FM101-treated groups during week 4 of dosing or week 2 of recovery. These lesions were also considered incidental due to a lack of dose-dependency.

In conclusion, FM101, an A_3_AR modulator, has been identified as a biased ligand that acts as a G protein agonist and β-arrestin antagonist. In this study, we demonstrated the toxicological potential of FM101 in rodents. The compound did not induce significant toxic changes, and we believe that the approximate lethal dose in rats is likely greater than the maximum dose of 2000 mg/kg tested in this study. Chronic exposure of up to 1000 mg/kg/day over 28 days indicates a no observed adverse effect level (NOAEL) of 1000 mg/kg/day for male and female rats. These findings support the further investigation of FM101 in clinical trials. However, we did not evaluate the toxicological profile of FM101 in non-rodent animals or humans, and differences between its effects and could only be determined using *in vitro *assessments. Further studies should address these concerns.

## Acknowledgements

These studies were supported by Futuremedicine Co. Ltd, Republic of Korea and a grant of the Korea health technology R&D Project in the Korea Health Industry Development Institute by Korean government (HI17C2262). We would like to thank the study sponsor and the funding sources for making this work possible. We would also like to thank Editage (www.editage.co.kr) for English language editing. 

## Supplementary Material

Supplementary data

## Figures and Tables

**Table 1:**
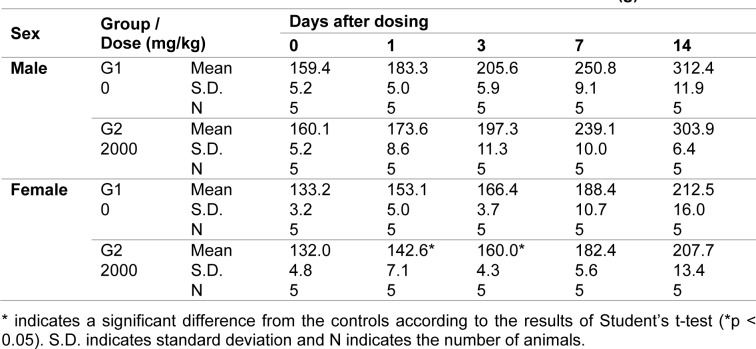
Summary results of body weight during the acute toxicity study

**Table 2:**
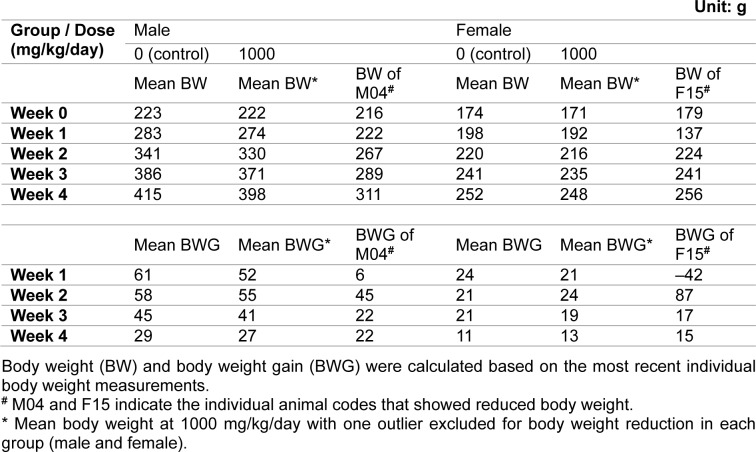
Summary of body weights and body weight gain during the subchronic toxicity study

**Table 3:**
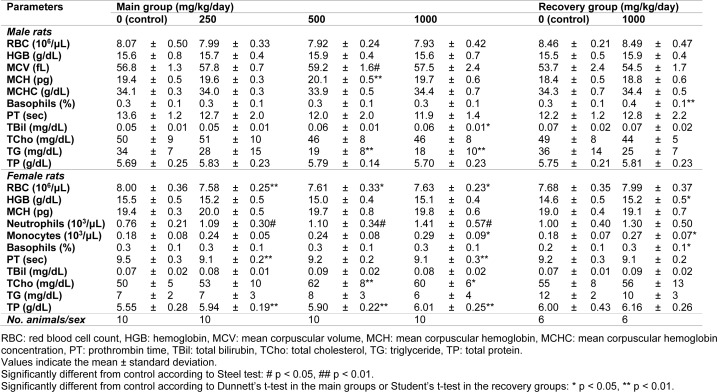
Summary of hematology and clinical pathology parameters in the subchronic toxicity study

**Table 4:**
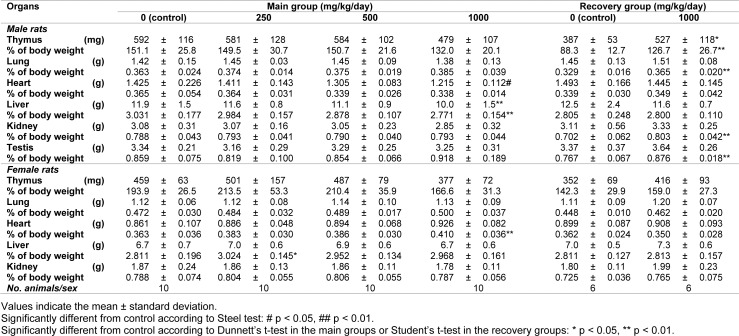
Summary of absolute and relative organ weights in the subchronic toxicity study

**Table 5:**
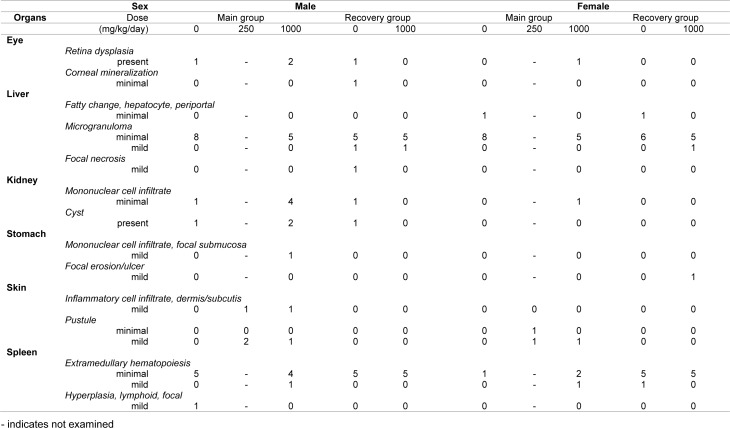
Incidence and severity of microscopic findings in the subchronic toxicity study
