# Supplementary material for: Safety evaluation of FM101, an A3 adenosine receptor modulator, in rat, for developing as therapeutics of glaucoma and hepatitis
Source: EXCLI J. 2020 Feb 12;19:187–200. doi: 10.17179/excli2019-2058 (PMC7105940; doi:10.17179/excli2019-2058)
Supplement: Supplementary data [file EXCLI-19-187-s-001.pdf]

EXCLI Journal 2020;19:187-200 – ISSN 1611-2156
Received: December 12, 2019, accepted: February 05, 2020, published: February 12, 2020

Supplementary data to:

SAFETY EVALUATION OF FM101, AN A3 ADENOSINE RECEPTOR MODULATOR, IN RAT, FOR DEVELOPING AS THERAPEUTICS OF GLAUCOMA AND HEPATITIS

Chong-Woo Park[1,2], Chung-Tack Han[3], Yasue Sakaguchi[4], Jiyoun Lee[1], Hwa-young Youn*[2]

[1] R&D Center, Futuremedicine Co., Ltd., Seongnam, Republic of Korea
[2] Laboratory of Veterinary Internal Medicine, College of Veterinary Medicine, Seoul National University, Seoul, Republic of Korea
[3] Biotoxtech Co., Ltd., Cheongju, Republic of Korea
[4] Ina Research Inc., Ina, Japan

* Corresponding author: Hwa-young Youn, College of Veterinary Medicine, Seoul National University, Seoul, Republic of Korea, Telephone number: 82-2-880-1266, Fax number: 82-31-757-2738, E-mail: hyyoun@snu.ac.kr

http://dx.doi.org/10.17179/excli2019-2058

This is an Open Access article distributed under the terms of the Creative Commons Attribution License (http://creativecommons.org/licenses/by/4.0/).

Supplementary Table 1: Raw data of individual body weight for acute toxicity study. The below raw data are related to **Table 1**.

Sex: Male | Unit: (g)

Group / Dose (mg/kg)	Animal ID	Days after dosing: 0	1	3	7	14
G1	M01	161.9	185.3	206.5	251.8	318.9
0 (control)	M02	166.7	189.0	213.3	258.0	327.4
	M03	157.7	185.9	207.3	259.9	312.8
	M04	158.1	179.5	203.6	246.9	307.0
	M05	152.8	176.9	197.1	237.3	296.0
	**Mean**	**159.4**	**183.3**	**205.6**	**250.8**	**312.4**
	**S.D.**	**5.2**	**5.0**	**5.9**	**9.1**	**11.9**
	**N**	**5**	**5**	**5**	**5**	**5**
G2	M01	162.3	178.4	205.7	245.4	305.4
2,000	M02	155.6	165.3	183.4	229.4	306.3
	M03	157.5	164.0	189.2	228.8	295.4
	M04	156.7	176.6	197.5	240.3	300.3
	M05	168.2	183.6	210.8	251.6	312.2
	**Mean**	**160.1**	**173.6**	**197.3**	**239.1**	**303.9**
	**S.D.**	**5.2**	**8.6**	**11.3**	**10.0**	**6.4**
	**N**	**5**	**5**	**5**	**5**	**5**

S.D. indicates standard deviation and N indicates number of animals.

EXCLI Journal 2020;19:187-200 – ISSN 1611-2156
Received: December 12, 2019, accepted: February 05, 2020, published: February 12, 2020

Supplementary Table 1 (cont.): Raw data of individual body weight for acute toxicity study. The below raw data are related to **Table 1**.

Sex: Female Unit: (g)

Group / Dose (mg/kg)	Animal ID	Days after dosing 0	1	3	7	14
G1	F01	133.7	155.6	167.6	192.2	230.7
0 (control)	F02	131.5	149.1	166.8	186.0	197.7
	F03	138.0	159.5	170.3	197.0	225.8
	F04	129.3	147.2	160.2	170.9	195.2
	F05	133.4	154.3	167.3	196.0	213.2
	**Mean**	**133.2**	**153.1**	**166.4**	**188.4**	**212.5**
	**S.D.**	**3.2**	**5.0**	**3.7**	**10.7**	**16.0**
	**N**	**5**	**5**	**5**	**5**	**5**
G2	F01	133.4	142.0	158.8	182.8	203.4
2,000	F02	131.9	143.4	158.9	185.0	213.1
	F03	129.9	149.7	162.0	185.1	205.2
	F04	125.8	131.1	154.3	172.7	190.3
	F05	139.0	146.6	165.9	186.6	226.6
	**Mean**	**132.0**	**142.6**	**160.0**	**182.4**	**207.7**
	**S.D.**	**4.8**	**7.1**	**4.3**	**5.6**	**13.4**
	**N**	**5**	**5**	**5**	**5**	**5**

S.D. indicates standard deviation and N indicates number of animals.

EXCLI Journal 2020;19:187-200 – ISSN 1611-2156
Received: December 12, 2019, accepted: February 05, 2020, published: February 12, 2020

Supplementary Table 2: Raw data of individual body weight for subacute toxicity study. The below raw data are related to **Table 2**.

Sex: Male Unit : (g)

Group / Dose (mg/kg/day)	Animal ID	Week						
		Dosing period					Recovery period	
		0	1	2	3	4	5	6
G1 0 (control)	M01	215	276	349	401	430		
	M02	228	283	340	370	392		
	M03	219	274	330	379	409		
	M04	223	291	362	416	445		
	M05	234	305	371	431	465		
	M06	228	295	356	413	440		
	M07	219	288	342	385	413		
	M08	223	285	339	374	400		
	M09	239	312	389	446	484		
	M10	210	254	291	326	347		
	M11	218	276	317	357	380	423	441
	M12	210	259	303	345	382	423	449
	M13	211	263	306	336	359	394	410
	M14	232	304	382	433	475	527	556
	M15	230	288	347	400	435	475	503
	M16	226	282	335	367	391	421	448
	**Mean**	**223**	**283**	**341**	**386**	**415**	**444**	**468**
	**S.D.**	**9**	**16**	**28**	**36**	**40**	**48**	**53**
	**N**	**16**	**16**	**16**	**16**	**16**	**6**	**6**

S.D. indicates standard deviation and N indicates number of animals.

EXCLI Journal 2020;19:187-200 – ISSN 1611-2156
Received: December 12, 2019, accepted: February 05, 2020, published: February 12, 2020

Supplementary Table 2 (cont.): Raw data of individual body weight for subacute toxicity study. The below raw data are related to **Table 2**.

Sex: Male Unit : (g)

Group / Dose (mg/kg/day)	Animal ID	Week						
		Dosing period					Recovery period	
		0	1	2	3	4	5	6
G2 250	M01	214	281	343	393	420		
	M02	225	277	334	375	405		
	M03	226	299	360	408	437		
	M04	227	282	340	377	405		
	M05	222	282	326	363	390		
	M06	220	281	339	384	408		
	M07	223	291	354	406	434		
	M08	220	284	344	392	424		
	M09	216	265	321	368	392		
	M10	220	283	357	411	446		
	**Mean**	**221**	**283**	**342**	**388**	**416**		
	**S.D.**	**4**	**9**	**13**	**17**	**19**		
	**N**	**10**	**10**	**10**	**10**	**10**		
G3 500	M01	225	284	344	383	412		
	M02	222	289	351	401	422		
	M03	214	253	304	336	361		
	M04	221	276	330	380	411		
	M05	222	283	344	382	408		
	M06	228	294	347	392	424		
	M07	228	284	348	396	434		
	M08	230	289	352	402	436		
	M09	229	295	369	421	464		
	M10	229	276	338	386	417		
	**Mean**	**225**	**282**	**343**	**388**	**419**		
	**S.D.**	**5**	**12**	**17**	**22**	**26**		
	**N**	**10**	**10**	**10**	**10**	**10**		

S.D. indicates standard deviation and N indicates number of animals.

EXCLI Journal 2020;19:187-200 – ISSN 1611-2156
Received: December 12, 2019, accepted: February 05, 2020, published: February 12, 2020

Supplementary Table 2 (cont.): Raw data of individual body weight for subacute toxicity study. The below raw data are related to **Table 2**.

Sex: Male Unit : (g)

Group / Dose (mg/kg/day)	Animal ID	Week						
		Dosing period					Recovery period	
		0	1	2	3	4	5	6
G4 1,000	M01	232	290	352	384	414		
	M02	221	283	339	379	410		
	M03	232	282	355	406	442		
	M04	216	222	267	289	311		
	M05	214	262	312	364	397		
	M06	219	267	298	335	361		
	M07	223	277	341	384	411		
	M08	228	278	325	345	374		
	M09	218	280	342	393	434		
	M10	221	263	319	357	382		
	M11	212	257	303	344	372	416	436
	M12	210	260	319	362	387	418	437
	M13	221	272	323	364	374	411	426
	M14	235	286	356	406	430	461	489
	M15	218	275	326	362	387	412	430
	M16	226	283	336	378	400	436	462
	**Mean**	**222**	**271**	**326**	**366**	**393**	**426**	**447**
	**S.D.**	**7**	**16**	**23**	**29**	**32**	**20**	**24**
	**N**	**16**	**16**	**16**	**16**	**16**	**6**	**6**

S.D. indicates standard deviation and N indicates number of animals.

EXCLI Journal 2020;19:187-200 – ISSN 1611-2156
Received: December 12, 2019, accepted: February 05, 2020, published: February 12, 2020

Supplementary Table 2 (cont.): Raw data of individual body weight for subacute toxicity study. The below raw data are related to **Table 2**.

Sex: Female Unit : (g)

Group / Dose (mg/kg/day)	Animal ID	Week						
		Dosing period					Recovery period	
		0	1	2	3	4	5	6
G1	F01	178	200	217	232	252		
0 (control)	F02	185	204	232	256	266		
	F03	170	215	240	259	278		
	F04	166	200	222	241	254		
	F05	173	202	215	233	253		
	F06	180	212	230	258	270		
	F07	174	201	215	244	254		
	F08	185	199	232	256	261		
	F09	179	198	223	242	251		
	F10	170	182	208	227	231		
	F11	156	172	183	206	212	221	229
	F12	169	196	220	234	248	262	265
	F13	184	206	232	254	265	283	301
	F14	173	201	218	244	253	266	271
	F15	176	194	219	240	242	253	263
	F16	167	193	209	232	243	263	270
	**Mean**	**174**	**198**	**220**	**241**	**252**	**258**	**267**
	**S.D.**	**8**	**10**	**13**	**14**	**16**	**21**	**23**
	**N**	**16**	**16**	**16**	**16**	**16**	**6**	**6**

S.D. indicates standard deviation and N indicates number of animals.

EXCLI Journal 2020;19:187-200 – ISSN 1611-2156
Received: December 12, 2019, accepted: February 05, 2020, published: February 12, 2020

Supplementary Table 2 (cont.): Raw data of individual body weight for subacute toxicity study. The below raw data are related to **Table 2**.

Sex: Female Unit : (g)

Group / Dose (mg/kg/day)	Animal ID	Week						
		Dosing period					Recovery period	
		0	1	2	3	4	5	6
G2 250	F01	171	184	197	227	237		
	F02	176	200	219	247	252		
	F03	180	198	226	242	253		
	F04	171	186	204	222	236		
	F05	183	214	244	266	291		
	F06	173	198	220	239	257		
	F07	168	192	209	227	249		
	F08	159	185	196	220	232		
	F09	170	196	215	234	246		
	F10	181	212	236	256	273		
	**Mean**	**173**	**197**	**217**	**238**	**253**		
	**S.D.**	**7**	**10**	**16**	**15**	**18**		
	**N**	**10**	**10**	**10**	**10**	**10**		
G3 500	F01	170	204	232	249	268		
	F02	168	198	228	241	266		
	F03	164	184	198	209	229		
	F04	171	206	233	251	266		
	F05	181	207	215	243	262		
	F06	165	182	199	212	228		
	F07	185	209	223	243	262		
	F08	175	197	221	245	258		
	F09	167	195	219	234	252		
	F10	174	207	234	249	265		
	**Mean**	**172**	**199**	**220**	**238**	**256**		
	**S.D.**	**7**	**10**	**13**	**15**	**15**		
	**N**	**10**	**10**	**10**	**10**	**10**		

S.D. indicates standard deviation and N indicates number of animals.

EXCLI Journal 2020;19:187-200 – ISSN 1611-2156
Received: December 12, 2019, accepted: February 05, 2020, published: February 12, 2020

Supplementary Table 2 (cont.): Raw data of individual body weight for subacute toxicity study. The below raw data are related to **Table 2**.

Sex: Female Unit : (g)

Group / Dose (mg/kg/day)	Animal ID	Week						
		Dosing period					Recovery period	
		0	1	2	3	4	5	6
G4	F01	162	187	206	221	233		
1,000	F02	169	187	214	230	236		
	F03	163	178	203	226	230		
	F04	172	182	211	227	235		
	F05	166	179	208	224	238		
	F06	182	201	241	261	271		
	F07	174	198	219	238	261		
	F08	169	191	214	234	236		
	F09	163	184	205	226	247		
	F10	177	196	220	246	259		
	F11	169	197	216	240	257	275	292
	F12	159	179	193	206	225	273	285
	F13	186	216	232	259	274	239	245
	F14	178	205	223	236	259	293	301
	F15	179	137	224	241	256	265	266
	F16	181	199	228	248	263	288	296
	**Mean**	**172**	**189**	**216**	**235**	**249**	**272**	**281**
	**S.D.**	**8**	**17**	**12**	**14**	**16**	**19**	**21**
	**N**	**16**	**16**	**16**	**16**	**16**	**6**	**6**

S.D. indicates standard deviation and N indicates number of animals.

EXCLI Journal 2020;19:187-200 – ISSN 1611-2156
Received: December 12, 2019, accepted: February 05, 2020, published: February 12, 2020

Supplementary Table 3-1: Raw data of hematological parameters after 4 weeks of dosing in main groups for subchronic toxicity study. The raw data are related to **Table 3**.

Sex: Male

Group / Dose (mg/kg/day)	Animal ID	RBC (×10^6 /μL)	HGB (g/dL)	RBC Indices MCV (fL)	MCH (pg)	MCHC (g/dL)	PT (sec)
G1 0 (control)	M01	7.31	14.4	58.3	19.7	33.8	13.9
	M02	8.38	15.6	55.2	18.6	33.6	13.2
	M03	8.01	15.4	56.7	19.3	34.0	12.7
	M04	7.40	14.5	57.6	19.6	34.1	11.6
	M05	7.50	14.8	56.9	19.7	34.7	12.1
	M06	8.09	16.3	58.6	20.1	34.3	15.0
	M07	8.45	16.0	55.6	18.9	33.9	14.0
	M08	8.26	16.1	57.3	19.5	34.0	15.4
	M09	8.48	16.4	57.1	19.3	33.8	13.9
	M10	8.77	16.5	54.7	18.9	34.5	14.4
	**Mean**	**8.07**	**15.6**	**56.8**	**19.4**	**34.1**	**13.6**
	**S.D.**	**0.50**	**0.8**	**1.3**	**0.5**	**0.3**	**1.2**
	**N**	**10**	**10**	**10**	**10**	**10**	**10**
G2 250	M01	7.87	15.4	58.2	19.5	33.5	11.5
	M02	8.24	16.1	57.1	19.5	34.1	10.9
	M03	7.78	15.5	58.5	19.9	34.0	15.2
	M04	7.72	15.3	58.1	19.8	34.1	12.4
	M05	7.82	15.5	58.2	19.8	34.0	11.5
	M06	8.76	16.7	56.6	19.0	33.7	11.0
	M07	7.62	15.3	58.5	20.1	34.4	10.7
	M08	7.94	15.6	57.1	19.7	34.5	15.8
	M09	8.10	16.0	58.5	19.8	33.8	12.4
	M10	8.08	15.6	57.3	19.3	33.7	15.1
	**Mean**	**7.99**	**15.7**	**57.8**	**19.6**	**34.0**	**12.7**
	**S.D.**	**0.33**	**0.4**	**0.7**	**0.3**	**0.3**	**2.0**
	**N**	**10**	**10**	**10**	**10**	**10**	**10**

RBC: red blood cell count, HGB: hemoglobin, MCV: mean corpuscular volume, MCH: mean corpuscular hemoglobin, MCHC: mean corpuscular hemoglobin concentration, PT: prothrombin time.

S.D. indicates standard deviation and N indicates number of animals.

EXCLI Journal 2020;19:187-200 – ISSN 1611-2156
Received: December 12, 2019, accepted: February 05, 2020, published: February 12, 2020

Supplementary Table 3-1 (cont.): Raw data of hematological parameters after 4 weeks of dosing in main groups for subchronic toxicity study. The raw data are related to **Table 3**.

Sex: Male

Group / Dose (mg/kg/day)	Animal ID	RBC ($\times 10^6$ /μL)	HGB (g/dL)	RBC Indices MCV (fL)	RBC Indices MCH (pg)	RBC Indices MCHC (g/dL)	PT (sec)
G3	M01	7.91	15.8	60.6	20.0	33.1	10.9
500	M02	8.18	16.4	60.3	20.1	33.3	9.9
	M03	7.69	15.3	58.5	19.9	34.1	15.6
	M04	7.62	15.8	61.0	20.7	33.9	12.8
	M05	8.07	15.8	56.4	19.6	34.7	10.9
	M06	8.19	16.3	57.8	19.9	34.4	15.3
	M07	8.21	16.2	58.6	19.8	33.7	10.7
	M08	7.63	16.1	61.5	21.1	34.3	11.8
	M09	7.74	15.4	58.2	19.9	34.2	10.4
	M10	7.96	15.9	59.3	20.0	33.6	11.9
	**Mean**	**7.92**	**15.9**	**59.2**	**20.1**	**33.9**	**12.0**
	**S.D.**	**0.24**	**0.4**	**1.6**	**0.5**	**0.5**	**2.0**
	**N**	**10**	**10**	**10**	**10**	**10**	**10**
G4	M01	7.97	15.7	58.5	19.7	33.8	12.7
1,000	M02	7.79	15.2	58.1	19.5	33.6	10.4
	M03	7.19	14.8	59.7	20.6	34.5	9.9
	M04	7.83	15.1	53.3	19.3	36.2	11.2
	M05	7.68	14.8	55.7	19.2	34.5	10.4
	M06	8.77	16.5	55.0	18.8	34.3	13.9
	M07	8.28	16.1	56.6	19.5	34.4	11.8
	M08	7.65	15.6	59.1	20.4	34.5	13.1
	M09	8.03	16.5	61.1	20.6	33.7	13.0
	M10	8.12	16.1	57.8	19.8	34.3	12.9
	**Mean**	**7.93**	**15.6**	**57.5**	**19.7**	**34.4**	**11.9**
	**S.D.**	**0.42**	**0.7**	**2.4**	**0.6**	**0.7**	**1.4**
	**N**	**10**	**10**	**10**	**10**	**10**	**10**

RBC: red blood cell count, HGB: hemoglobin, MCV: mean corpuscular volume, MCH: mean corpuscular hemoglobin, MCHC: mean corpuscular hemoglobin concentration, PT: prothrombin time.

S.D. indicates standard deviation and N indicates number of animals.

EXCLI Journal 2020;19:187-200 – ISSN 1611-2156
Received: December 12, 2019, accepted: February 05, 2020, published: February 12, 2020

Supplementary Table 3-1 (cont.): Raw data of hematological parameters after 4 weeks of dosing in main groups for subchronic toxicity study. The raw data are related to **Table 3**.

Sex: Male

Group / Dose (mg/kg/day)	Animal ID	WBC (×10³ /µL)	WBC differential count					
			Ratio (%)			Absolute count (×10³/µL)		
			NEU	MONO	BASO	NEU	MONO	BASO
G1	M01	12.60	15.4	2.5	0.4	1.94	0.31	0.05
0 (control)	M02	5.86	20.4	2.2	0.2	1.20	0.13	0.01
	M03	6.62	10.8	1.6	0.3	0.72	0.11	0.02
	M04	13.77	12.4	3.2	0.3	1.71	0.44	0.05
	M05	15.70	18.9	4.4	0.4	2.97	0.69	0.06
	M06	14.69	9.5	3.4	0.3	1.40	0.50	0.05
	M07	12.06	8.2	4.4	0.3	0.99	0.53	0.03
	M08	13.51	8.2	3.5	0.3	1.11	0.48	0.04
	M09	9.97	11.4	4.2	0.4	1.13	0.42	0.04
	M10	11.04	11.8	2.2	0.2	1.31	0.24	0.03
	**Mean**	**11.58**	**12.7**	**3.2**	**0.3**	**1.45**	**0.39**	**0.04**
	**S.D.**	**3.28**	**4.2**	**1.0**	**0.1**	**0.64**	**0.18**	**0.02**
	**N**	**10**	**10**	**10**	**10**	**10**	**10**	**10**
G2	M01	11.92	18.9	3.0	0.3	2.25	0.36	0.03
250	M02	9.09	15.5	2.5	0.3	1.41	0.22	0.03
	M03	15.59	11.5	3.2	0.4	1.79	0.50	0.06
	M04	12.52	11.7	1.9	0.3	1.46	0.24	0.04
	M05	9.81	17.2	1.6	0.3	1.68	0.16	0.03
	M06	16.43	12.5	1.8	0.5	2.05	0.29	0.08
	M07	11.33	17.3	4.2	0.2	1.96	0.47	0.03
	M08	13.62	7.7	2.0	0.2	1.05	0.27	0.03
	M09	8.80	10.5	4.3	0.3	0.93	0.38	0.03
	M10	11.72	22.6	3.3	0.3	2.65	0.39	0.04
	**Mean**	**12.08**	**14.5**	**2.8**	**0.3**	**1.72**	**0.33**	**0.04**
	**S.D.**	**2.57**	**4.5**	**1.0**	**0.1**	**0.53**	**0.11**	**0.02**
	**N**	**10**	**10**	**10**	**10**	**10**	**10**	**10**

WBC: white blood cells, NEU: neutrophils, MONO: monocytes, BASO: basophils.

S.D. indicates standard deviation and N indicates number of animals.

EXCLI Journal 2020;19:187-200 – ISSN 1611-2156
Received: December 12, 2019, accepted: February 05, 2020, published: February 12, 2020

Supplementary Table 3-1 (cont.): Raw data of hematological parameters after 4 weeks of dosing in main groups for subchronic toxicity study. The raw data are related to **Table 3**.

Sex: Male

Group / Dose (mg/kg/day)	Animal ID	WBC (×10³ /µL)	WBC differential count Ratio (%) NEU	Ratio (%) MONO	Ratio (%) BASO	Absolute count (×10³/µL) NEU	Absolute count (×10³/µL) MONO	Absolute count (×10³/µL) BASO
G3	M01	8.54	28.6	2.4	0.2	2.44	0.20	0.02
500	M02	8.15	22.3	4.0	0.4	1.82	0.33	0.03
	M03	7.40	15.8	1.7	0.2	1.17	0.13	0.01
	M04	11.53	19.0	2.0	0.3	2.19	0.23	0.04
	M05	6.34	16.7	3.1	0.2	1.06	0.20	0.01
	M06	11.07	9.6	3.4	0.3	1.06	0.38	0.03
	M07	8.73	11.7	2.3	0.2	1.02	0.20	0.02
	M08	10.34	13.0	3.2	0.4	1.34	0.33	0.04
	M09	9.70	12.1	3.7	0.3	1.18	0.36	0.03
	M10	9.85	17.4	2.6	0.4	1.71	0.25	0.04
	**Mean**	**9.17**	**16.6**	**2.8**	**0.3**	**1.50**	**0.26**	**0.03**
	**S.D.**	**1.64**	**5.7**	**0.8**	**0.1**	**0.51**	**0.08**	**0.01**
	**N**	**10**	**10**	**10**	**10**	**10**	**10**	**10**
G4	M01	10.12	10.7	2.1	0.2	1.08	0.22	0.02
1,000	M02	15.34	13.3	2.2	0.4	2.04	0.33	0.06
	M03	12.90	20.1	4.4	0.3	2.59	0.57	0.04
	M04	6.59	20.1	3.2	0.2	1.32	0.21	0.02
	M05	11.18	16.7	1.2	0.3	1.87	0.13	0.03
	M06	9.74	9.2	1.6	0.2	0.90	0.16	0.02
	M07	9.84	16.0	5.6	0.3	1.57	0.55	0.03
	M08	13.40	10.9	1.9	0.3	1.47	0.25	0.04
	M09	11.10	12.7	4.0	0.3	1.41	0.44	0.03
	M10	8.64	11.6	2.4	0.3	1.01	0.20	0.03
	**Mean**	**10.89**	**14.1**	**2.9**	**0.3**	**1.53**	**0.31**	**0.03**
	**S.D.**	**2.51**	**3.9**	**1.4**	**0.1**	**0.52**	**0.16**	**0.01**
	**N**	**10**	**10**	**10**	**10**	**10**	**10**	**10**

WBC: white blood cells, NEU: neutrophils, MONO: monocytes, BASO: basophils.

S.D. indicates standard deviation and N indicates number of animals.

EXCLI Journal 2020;19:187-200 – ISSN 1611-2156
Received: December 12, 2019, accepted: February 05, 2020, published: February 12, 2020

Supplementary Table 3-1 (cont.): Raw data of hematological parameters after 4 weeks of dosing in main groups for subchronic toxicity study. The raw data are related to **Table 3**.

Sex: Female

Group / Dose (mg/kg/day)	Animal ID	RBC ($\times 10^6$ /μL)	HGB (g/dL)	RBC Indices MCV (fL)	MCH (pg)	MCHC (g/dL)	PT (sec)
G1	F01	8.53	16.2	55.5	18.9	34.1	10.1
0 (control)	F02	7.93	15.5	55.0	19.5	35.5	9.7
	F03	7.72	15.2	55.2	19.7	35.6	9.3
	F04	7.62	14.8	55.0	19.5	35.4	9.2
	F05	7.56	14.8	56.2	19.6	34.9	9.9
	F06	7.90	15.7	57.8	19.9	34.5	9.4
	F07	8.51	16.2	54.4	19.0	35.0	9.3
	F08	7.78	15.1	55.8	19.5	34.9	9.3
	F09	8.15	15.8	54.8	19.4	35.5	9.5
	F10	8.33	15.9	53.0	19.1	36.0	9.3
	**Mean**	**8.00**	**15.5**	**55.3**	**19.4**	**35.1**	**9.5**
	**S.D.**	**0.36**	**0.5**	**1.2**	**0.3**	**0.6**	**0.3**
	**N**	**10**	**10**	**10**	**10**	**10**	**10**
G2	F01	8.04	15.3	53.8	19.0	35.4	9.3
250	F02	7.49	14.8	55.8	19.8	35.4	8.8
	F03	7.37	15.3	58.4	20.7	35.5	8.8
	F04	7.36	14.4	56.1	19.6	35.0	8.9
	F05	7.79	15.7	57.4	20.1	35.1	8.9
	F06	7.75	15.5	56.9	20.0	35.2	9.2
	F07	7.73	15.9	57.9	20.5	35.5	9.4
	F08	7.65	15.5	57.5	20.3	35.2	9.3
	F09	7.39	14.7	57.8	20.0	34.5	9.1
	F10	7.25	14.7	57.8	20.2	35.0	9.3
	**Mean**	**7.58**	**15.2**	**56.9**	**20.0**	**35.2**	**9.1**
	**S.D.**	**0.25**	**0.5**	**1.4**	**0.5**	**0.3**	**0.2**
	**N**	**10**	**10**	**10**	**10**	**10**	**10**

RBC: red blood cell count, HGB: hemoglobin, MCV: mean corpuscular volume, MCH: mean corpuscular hemoglobin, MCHC: mean corpuscular hemoglobin concentration, PT: prothrombin time.

S.D. indicates standard deviation and N indicates number of animals.

EXCLI Journal 2020;19:187-200 – ISSN 1611-2156
Received: December 12, 2019, accepted: February 05, 2020, published: February 12, 2020

Supplementary Table 3-1 (cont.): Raw data of hematological parameters after 4 weeks of dosing in main groups for subchronic toxicity study. The raw data are related to **Table 3**.

Sex: Female

Group / Dose (mg/kg/day)	Animal ID	RBC ($\times 10^6$ /μL)	HGB (g/dL)	RBC Indices			PT (sec)
				MCV (fL)	MCH (pg)	MCHC (g/dL)	
G3 500	F01	7.27	15.2	59.9	20.9	34.9	9.4
	F02	7.25	15.4	60.0	21.3	35.5	9.6
	F03	8.21	15.6	55.8	19.0	34.1	9.3
	F04	7.71	15.1	55.4	19.6	35.4	8.9
	F05	7.98	15.3	56.2	19.2	34.2	9.0
	F06	7.61	15.0	56.0	19.8	35.3	9.1
	F07	7.76	14.5	54.6	18.7	34.3	9.2
	F08	7.60	14.7	56.2	19.3	34.4	9.3
	F09	7.18	14.3	58.1	19.9	34.3	9.3
	F10	7.53	14.8	55.7	19.7	35.3	9.3
	**Mean**	**7.61**	**15.0**	**56.8**	**19.7**	**34.8**	**9.2**
	**S.D.**	**0.33**	**0.4**	**1.9**	**0.8**	**0.6**	**0.2**
	**N**	**10**	**10**	**10**	**10**	**10**	**10**
G4 1,000	F01	7.91	15.5	56.2	19.5	34.7	9.7
	F02	7.20	15.0	59.1	20.9	35.3	9.2
	F03	7.56	15.1	56.6	20.0	35.3	8.8
	F04	8.03	15.6	54.4	19.4	35.7	8.9
	F05	7.58	14.3	53.7	18.9	35.2	8.9
	F06	7.55	15.0	56.7	19.8	35.0	8.9
	F07	7.64	14.7	54.5	19.3	35.4	9.3
	F08	7.73	15.3	56.3	19.7	35.1	8.8
	F09	7.46	15.1	58.6	20.3	34.6	9.6
	F10	7.61	15.2	57.4	20.0	34.9	9.1
	**Mean**	**7.63**	**15.1**	**56.4**	**19.8**	**35.1**	**9.1**
	**S.D.**	**0.23**	**0.4**	**1.8**	**0.6**	**0.3**	**0.3**
	**N**	**10**	**10**	**10**	**10**	**10**	**10**

RBC: red blood cell count, HGB: hemoglobin, MCV: mean corpuscular volume, MCH: mean corpuscular hemoglobin, MCHC: mean corpuscular hemoglobin concentration, PT: prothrombin time.

S.D. indicates standard deviation and N indicates number of animals.

EXCLI Journal 2020;19:187-200 – ISSN 1611-2156
Received: December 12, 2019, accepted: February 05, 2020, published: February 12, 2020

Supplementary Table 3-1 (cont.): Raw data of hematological parameters after 4 weeks of dosing in main groups for subchronic toxicity study. The raw data are related to **Table 3**.

Sex: Female

Group / Dose (mg/kg/day)	Animal ID	WBC (×10^3 /μL)	WBC differential count					
			Ratio (%)			Absolute count (×10^3/μL)		
			NEU	MONO	BASO	NEU	MONO	BASO
G1	F01	5.86	11.2	2.2	0.3	0.66	0.13	0.02
0 (control)	F02	10.01	6.0	2.4	0.2	0.60	0.24	0.02
	F03	8.18	9.5	3.0	0.1	0.78	0.25	0.01
	F04	7.57	15.2	1.9	0.2	1.15	0.15	0.01
	F05	4.82	15.6	1.2	0.2	0.75	0.06	0.01
	F06	5.16	15.5	1.3	0.2	0.80	0.07	0.01
	F07	8.04	7.5	3.7	0.6	0.60	0.30	0.05
	F08	7.14	11.2	3.0	0.2	0.80	0.21	0.01
	F09	6.96	6.4	2.4	0.2	0.44	0.16	0.02
	F10	10.63	9.5	2.4	0.3	1.01	0.25	0.03
	**Mean**	**7.44**	**10.8**	**2.4**	**0.3**	**0.76**	**0.18**	**0.02**
	**S.D.**	**1.90**	**3.7**	**0.8**	**0.1**	**0.21**	**0.08**	**0.01**
	**N**	**10**	**10**	**10**	**10**	**10**	**10**	**10**
G2	F01	8.15	12.7	3.2	0.3	1.04	0.26	0.03
250	F02	8.45	8.4	1.9	0.2	0.71	0.16	0.02
	F03	7.02	12.5	2.5	0.2	0.88	0.17	0.02
	F04	8.73	17.8	3.1	0.2	1.56	0.27	0.02
	F05	11.01	8.5	2.0	0.3	0.93	0.22	0.03
	F06	6.39	13.1	3.0	0.3	0.83	0.19	0.02
	F07	8.78	11.1	2.9	0.2	0.98	0.26	0.02
	F08	9.25	11.6	3.0	0.3	1.08	0.28	0.03
	F09	12.39	10.9	2.1	0.2	1.35	0.26	0.03
	F10	9.06	17.4	3.2	0.3	1.58	0.29	0.02
	**Mean**	**8.92**	**12.4**	**2.7**	**0.3**	**1.09**	**0.24**	**0.02**
	**S.D.**	**1.75**	**3.2**	**0.5**	**0.1**	**0.30**	**0.05**	**0.01**
	**N**	**10**	**10**	**10**	**10**	**10**	**10**	**10**

WBC: white blood cells, NEU: neutrophils, MONO: monocytes, BASO: basophils.

S.D. indicates standard deviation and N indicates number of animals.

EXCLI Journal 2020;19:187-200 – ISSN 1611-2156
Received: December 12, 2019, accepted: February 05, 2020, published: February 12, 2020

Supplementary Table 3-1 (cont.): Raw data of hematological parameters after 4 weeks of dosing in main groups for subchronic toxicity study. The raw data are related to **Table 3**.

Sex: Female

Group / Dose (mg/kg/day)	Animal ID	WBC (×10³ /μL)	WBC differential count					
			Ratio (%)			Absolute count (×10³/μL)		
			NEU	MONO	BASO	NEU	MONO	BASO
G3 500	F01	9.39	18.0	3.7	0.3	1.70	0.35	0.03
	F02	8.56	18.2	2.3	0.2	1.56	0.20	0.02
	F03	4.32	17.8	2.8	0.3	0.77	0.12	0.01
	F04	7.05	16.0	3.5	0.3	1.13	0.25	0.02
	F05	4.73	17.1	2.2	0.2	0.81	0.10	0.01
	F06	9.32	9.7	3.8	0.4	0.91	0.35	0.03
	F07	10.24	13.1	2.3	0.2	1.34	0.24	0.02
	F08	12.08	6.9	2.3	0.4	0.83	0.28	0.04
	F09	9.72	7.7	2.5	0.3	0.75	0.24	0.03
	F10	10.71	10.9	2.7	0.3	1.17	0.29	0.03
	**Mean**	**8.61**	**13.5**	**2.8**	**0.3**	**1.10**	**0.24**	**0.02**
	**S.D.**	**2.52**	**4.5**	**0.6**	**0.1**	**0.34**	**0.08**	**0.01**
	**N**	**10**	**10**	**10**	**10**	**10**	**10**	**10**
G4 1,000	F01	5.82	25.7	3.4	0.2	1.50	0.20	0.01
	F02	8.92	11.2	3.3	0.3	1.00	0.29	0.03
	F03	7.23	5.8	3.0	0.3	0.42	0.22	0.02
	F04	12.42	10.7	2.5	0.4	1.33	0.31	0.05
	F05	8.89	17.5	2.3	0.3	1.56	0.20	0.02
	F06	9.82	16.3	4.8	0.3	1.60	0.48	0.03
	F07	11.71	20.5	3.1	0.2	2.39	0.36	0.03
	F08	11.39	6.2	2.8	0.3	0.71	0.32	0.03
	F09	8.95	19.4	2.6	0.3	1.74	0.23	0.03
	F10	9.78	18.4	2.6	0.3	1.80	0.26	0.03
	**Mean**	**9.49**	**15.2**	**3.0**	**0.3**	**1.41**	**0.29**	**0.03**
	**S.D.**	**2.02**	**6.5**	**0.7**	**0.1**	**0.57**	**0.09**	**0.01**
	**N**	**10**	**10**	**10**	**10**	**10**	**10**	**10**

WBC: white blood cells, NEU: neutrophils, MONO: monocytes, BASO: basophils.

S.D. indicates standard deviation and N indicates number of animals.

EXCLI Journal 2020;19:187-200 – ISSN 1611-2156
Received: December 12, 2019, accepted: February 05, 2020, published: February 12, 2020

Supplementary Table 3-2: Raw data of hematological parameters in recovery groups after 2 weeks of recovery period for subchronic toxicity study. The raw data are related to **Table 3**.

Sex: Male

Group / Dose (mg/kg/day)	Animal ID	RBC ($\times 10^6$ /μL)	HGB (g/dL)	RBC Indices MCV (fL)	MCH (pg)	MCHC (g/dL)	PT (sec)
G1	M11	8.78	16.0	53.7	18.3	34.0	11.7
0 (control)	M12	8.29	15.8	56.7	19.1	33.6	12.2
	M13	8.52	15.6	54.3	18.4	33.8	10.8
	M14	8.60	15.9	53.0	18.5	35.0	11.5
	M15	8.21	15.2	54.8	18.5	33.8	12.7
	M16	8.38	14.7	49.6	17.6	35.4	14.4
	**Mean**	**8.46**	**15.5**	**53.7**	**18.4**	**34.3**	**12.2**
	**S.D.**	**0.21**	**0.5**	**2.4**	**0.5**	**0.7**	**1.2**
	**N**	**6**	**6**	**6**	**6**	**6**	**6**
G4	M11	8.48	15.6	53.9	18.4	34.2	13.6
1,000	M12	8.04	15.4	55.5	19.2	34.6	12.7
	M13	8.50	16.1	56.0	18.9	33.8	10.6
	M14	8.19	15.7	55.9	19.2	34.3	10.6
	M15	9.37	16.6	51.4	17.7	34.5	16.5
	M16	8.36	16.0	54.3	19.1	35.2	12.6
	**Mean**	**8.49**	**15.9**	**54.5**	**18.8**	**34.4**	**12.8**
	**S.D.**	**0.47**	**0.4**	**1.7**	**0.6**	**0.5**	**2.2**
	**N**	**6**	**6**	**6**	**6**	**6**	**6**

RBC: red blood cell count, HGB: hemoglobin, MCV: mean corpuscular volume, MCH: mean corpuscular hemoglobin, MCHC: mean corpuscular hemoglobin concentration, PT: prothrombin time.

S.D. indicates standard deviation and N indicates number of animals.

EXCLI Journal 2020;19:187-200 – ISSN 1611-2156
Received: December 12, 2019, accepted: February 05, 2020, published: February 12, 2020

Supplementary Table 3-2 (cont.): Raw data of hematological parameters in recovery groups after 2 weeks of recovery period for subchronic toxicity study. The raw data are related to **Table 3**.

Sex: Male

Group / Dose (mg/kg/day)	Animal ID	WBC (×10^3 /μL)	WBC differential count					
			Ratio (%)			Absolute count (×10^3/μL)		
			NEU	MONO	BASO	NEU	MONO	BASO
G1 0 (control)	M11	11.06	24.2	2.7	0.2	2.67	0.30	0.03
	M12	7.00	15.9	4.6	0.2	1.11	0.32	0.01
	M13	12.92	14.6	3.2	0.3	1.88	0.41	0.04
	M14	13.10	15.1	4.4	0.3	1.98	0.58	0.04
	M15	12.67	12.2	4.0	0.3	1.55	0.51	0.04
	M16	10.14	16.8	2.9	0.2	1.71	0.30	0.02
	**Mean**	**11.15**	**16.5**	**3.6**	**0.3**	**1.82**	**0.40**	**0.03**
	**S.D.**	**2.35**	**4.1**	**0.8**	**0.1**	**0.52**	**0.12**	**0.01**
	**N**	**6**	**6**	**6**	**6**	**6**	**6**	**6**
G4 1,000	M11	12.36	17.1	4.4	0.4	2.11	0.54	0.05
	M12	10.35	20.0	3.9	0.3	2.07	0.41	0.03
	M13	16.05	10.9	2.6	0.4	1.75	0.42	0.06
	M14	16.19	13.8	3.1	0.4	2.23	0.51	0.07
	M15	7.51	14.2	2.9	0.3	1.07	0.22	0.03
	M16	9.43	29.8	2.2	0.4	2.81	0.21	0.04
	**Mean**	**11.98**	**17.6**	**3.2**	**0.4**	**2.01**	**0.39**	**0.05**
	**S.D.**	**3.57**	**6.7**	**0.8**	**0.1**	**0.58**	**0.14**	**0.02**
	**N**	**6**	**6**	**6**	**6**	**6**	**6**	**6**

WBC: white blood cells, NEU: neutrophils, MONO: monocytes, BASO: basophils.

S.D. indicates standard deviation and N indicates number of animals.

EXCLI Journal 2020;19:187-200 – ISSN 1611-2156
Received: December 12, 2019, accepted: February 05, 2020, published: February 12, 2020

Supplementary Table 3-2 (cont.): Raw data of hematological parameters in recovery groups after 2 weeks of recovery period for subchronic toxicity study. The raw data are related to **Table 3**.

Sex: Female

Group / Dose (mg/kg/day)	Animal ID	RBC ($\times 10^6$ /µL)	HGB (g/dL)	RBC Indices MCV (fL)	MCH (pg)	MCHC (g/dL)	PT (sec)
G1 0 (control)	F11	7.82	14.6	53.4	18.7	35.0	9.5
	F12	7.57	14.6	55.5	19.2	34.6	9.3
	F13	7.39	13.8	52.0	18.6	35.8	9.4
	F14	8.21	15.4	54.8	18.8	34.2	9.0
	F15	7.25	14.2	59.0	19.6	33.2	9.5
	F16	7.85	14.8	54.3	18.8	34.7	8.7
	**Mean**	**7.68**	**14.6**	**54.8**	**19.0**	**34.6**	**9.2**
	**S.D.**	**0.35**	**0.5**	**2.4**	**0.4**	**0.9**	**0.3**
	**N**	**6**	**6**	**6**	**6**	**6**	**6**
G4 1,000	F11	7.97	15.7	58.0	19.7	33.9	9.3
	F12	7.95	15.1	54.8	19.0	34.7	9.2
	F13	7.96	14.6	53.4	18.4	34.5	9.1
	F14	7.46	14.8	55.0	19.8	36.0	8.8
	F15	8.01	15.4	55.8	19.2	34.4	8.8
	F16	8.61	15.7	53.3	18.2	34.2	9.2
	**Mean**	**7.99**	**15.2**	**55.1**	**19.1**	**34.6**	**9.1**
	**S.D.**	**0.37**	**0.5**	**1.7**	**0.7**	**0.7**	**0.2**
	**N**	**6**	**6**	**6**	**6**	**6**	**6**

RBC: red blood cell count, HGB: hemoglobin, MCV: mean corpuscular volume, MCH: mean corpuscular hemoglobin, MCHC: mean corpuscular hemoglobin concentration, PT: prothrombin time.

S.D. indicates standard deviation and N indicates number of animals.

EXCLI Journal 2020;19:187-200 – ISSN 1611-2156
Received: December 12, 2019, accepted: February 05, 2020, published: February 12, 2020

Supplementary Table 3-2 (cont.): Raw data of hematological parameters in recovery groups after 2 weeks of recovery period for subchronic toxicity study. The raw data are related to **Table 3**.

Sex: Female

Group / Dose (mg/kg/day)	Animal ID	WBC (×10³ /μL)	WBC differential count					
			Ratio (%)			Absolute count (×10³/μL)		
			NEU	MONO	BASO	NEU	MONO	BASO
G1	F11	5.08	21.1	2.6	0.2	1.07	0.13	0.01
0 (control)	F12	7.12	16.0	4.1	0.2	1.14	0.29	0.02
	F13	5.99	9.1	2.3	0.1	0.54	0.14	0.01
	F14	6.54	25.6	3.4	0.2	1.68	0.22	0.01
	F15	5.21	15.1	2.1	0.1	0.79	0.11	0.00
	F16	6.33	12.5	2.5	0.3	0.79	0.16	0.02
	**Mean**	**6.05**	**16.6**	**2.8**	**0.2**	**1.00**	**0.18**	**0.01**
	**S.D.**	**0.79**	**5.9**	**0.8**	**0.1**	**0.40**	**0.07**	**0.01**
	**N**	**6**	**6**	**6**	**6**	**6**	**6**	**6**
G4	F11	10.62	17.6	3.3	0.3	1.87	0.35	0.03
1,000	F12	6.96	16.0	3.6	0.3	1.11	0.25	0.02
	F13	5.87	14.0	4.5	0.2	0.82	0.27	0.01
	F14	6.95	18.3	2.1	0.3	1.27	0.15	0.02
	F15	9.51	20.3	3.1	0.2	1.93	0.30	0.02
	F16	6.24	12.8	4.3	0.3	0.80	0.27	0.02
	**Mean**	**7.69**	**16.5**	**3.5**	**0.3**	**1.30**	**0.27**	**0.02**
	**S.D.**	**1.92**	**2.8**	**0.9**	**0.1**	**0.50**	**0.07**	**0.01**
	**N**	**6**	**6**	**6**	**6**	**6**	**6**	**6**

WBC: white blood cells, NEU: neutrophils, MONO: monocytes, BASO: basophils.

S.D. indicates standard deviation and N indicates number of animals.

EXCLI Journal 2020;19:187-200 – ISSN 1611-2156
Received: December 12, 2019, accepted: February 05, 2020, published: February 12, 2020

Supplementary Table 3-3: Raw data of clinical chemistry parameters after 4 weeks of dosing in main groups for subchronic toxicity study. The raw data are related to **Table 3**.

Sex: Male

Group / Dose (mg/kg/day)	Animal ID	TBili (mg/dL)	TCho (mg/dL)	TG (mg/dL)	TP (g/dL)
G1	M01	0.04	44	31	5.41
0 (control)	M02	0.05	32	25	5.55
	M03	0.05	49	28	5.99
	M04	0.04	52	29	5.71
	M05	0.05	65	45	5.79
	M06	0.05	62	37	6.04
	M07	0.04	56	31	5.89
	M08	0.05	51	32	5.49
	M09	0.05	47	48	5.70
	M10	0.06	45	34	5.29
	**Mean**	**0.05**	**50**	**34**	**5.69**
	**S.D.**	**0.01**	**9**	**7**	**0.25**
	**N**	**10**	**10**	**10**	**10**
G2	M01	0.05	53	27	6.11
250	M02	0.06	64	18	5.82
	M03	0.05	57	49	5.93
	M04	0.05	45	10	5.62
	M05	0.06	39	19	5.75
	M06	0.05	57	27	6.09
	M07	0.04	47	16	5.67
	M08	0.04	46	56	5.57
	M09	0.05	64	23	5.59
	M10	0.04	36	36	6.15
	**Mean**	**0.05**	**51**	**28**	**5.83**
	**S.D.**	**0.01**	**10**	**15**	**0.23**
	**N**	**10**	**10**	**10**	**10**

TBil: total bilirubin, TCho: total cholesterol, TG: triglyceride, TP: total protein.

S.D. indicates standard deviation and N indicates number of animals.

EXCLI Journal 2020;19:187-200 – ISSN 1611-2156
Received: December 12, 2019, accepted: February 05, 2020, published: February 12, 2020

Supplementary Table 3-3 (cont.): Raw data of clinical chemistry parameters after 4 weeks of dosing in main groups for subchronic toxicity study. The raw data are related to **Table 3**.

Sex: Male

Group / Dose (mg/kg/day)	Animal ID	TBili (mg/dL)	TCho (mg/dL)	TG (mg/dL)	TP (g/dL)
G3	M01	0.05	43	14	5.74
500	M02	0.05	49	21	5.96
	M03	0.07	33	9	5.59
	M04	0.06	51	26	5.96
	M05	0.08	37	15	5.90
	M06	0.06	39	34	5.76
	M07	0.05	54	14	5.64
	M08	0.05	59	21	5.74
	M09	0.05	49	25	5.95
	M10	0.05	45	9	5.67
	**Mean**	**0.06**	**46**	**19**	**5.79**
	**S.D.**	**0.01**	**8**	**8**	**0.14**
	**N**	**10**	**10**	**10**	**10**
G4	M01	0.05	43	14	5.69
1,000	M02	0.04	48	40	5.73
	M03	0.07	67	15	5.94
	M04	0.08	48	4	6.09
	M05	0.05	41	18	5.57
	M06	0.06	35	9	5.31
	M07	0.07	42	26	5.93
	M08	0.06	45	16	5.55
	M09	0.06	47	25	5.65
	M10	0.06	46	12	5.52
	**Mean**	**0.06**	**46**	**18**	**5.70**
	**S.D.**	**0.01**	**8**	**10**	**0.23**
	**N**	**10**	**10**	**10**	**10**

TBil: total bilirubin, TCho: total cholesterol, TG: triglyceride, TP: total protein.

S.D. indicates standard deviation and N indicates number of animals.

EXCLI Journal 2020;19:187-200 – ISSN 1611-2156
Received: December 12, 2019, accepted: February 05, 2020, published: February 12, 2020

Supplementary Table 3-3 (cont.): Raw data of clinical chemistry parameters after 4 weeks of dosing in main groups for subchronic toxicity study. The raw data are related to **Table 3**.

Sex: Female

Group / Dose (mg/kg/day)	Animal ID	TBili (mg/dL)	TCho (mg/dL)	TG (mg/dL)	TP (g/dL)
G1	F01	0.06	48	5	5.61
0 (control)	F02	0.07	50	10	5.34
	F03	0.04	54	8	5.93
	F04	0.06	49	10	5.18
	F05	0.08	41	4	5.30
	F06	0.05	45	7	5.57
	F07	0.10	51	8	6.06
	F08	0.07	48	8	5.31
	F09	0.10	57	5	5.56
	F10	0.05	55	9	5.61
	**Mean**	**0.07**	**50**	**7**	**5.55**
	**S.D.**	**0.02**	**5**	**2**	**0.28**
	**N**	**10**	**10**	**10**	**10**
G2	F01	0.06	42	5	5.98
250	F02	0.06	42	13	5.59
	F03	0.10	58	4	6.11
	F04	0.07	52	6	6.05
	F05	0.08	68	7	6.01
	F06	0.08	67	7	5.98
	F07	0.07	46	4	6.17
	F08	0.10	58	11	6.00
	F09	0.07	50	4	5.64
	F10	0.08	44	7	5.89
	**Mean**	**0.08**	**53**	**7**	**5.94**
	**S.D.**	**0.01**	**10**	**3**	**0.19**
	**N**	**10**	**10**	**10**	**10**

TBil: total bilirubin, TCho: total cholesterol, TG: triglyceride, TP: total protein.

S.D. indicates standard deviation and N indicates number of animals.

EXCLI Journal 2020;19:187-200 – ISSN 1611-2156
Received: December 12, 2019, accepted: February 05, 2020, published: February 12, 2020

Supplementary Table 3-3 (cont.): Raw data of clinical chemistry parameters after 4 weeks of dosing in main groups for subchronic toxicity study. The raw data are related to **Table 3**.

Sex: Female

Group / Dose (mg/kg/day)	Animal ID	TBili (mg/dL)	TCho (mg/dL)	TG (mg/dL)	TP (g/dL)
G3	F01	0.09	66	10	5.90
500	F02	0.07	50	10	5.74
	F03	0.08	60	6	5.69
	F04	0.07	62	7	6.13
	F05	0.09	65	5	6.27
	F06	0.12	57	8	6.05
	F07	0.07	73	14	5.65
	F08	0.07	70	7	5.98
	F09	0.08	64	9	5.62
	F10	0.11	51	6	5.98
	**Mean**	**0.09**	**62**	**8**	**5.90**
	**S.D.**	**0.02**	**8**	**3**	**0.22**
	**N**	**10**	**10**	**10**	**10**
G4	F01	0.09	59	7	5.97
1,000	F02	0.07	53	3	5.87
	F03	0.08	58	4	6.11
	F04	0.09	60	7	6.16
	F05	0.08	50	5	6.06
	F06	0.08	57	16	6.14
	F07	0.11	61	6	6.39
	F08	0.08	62	4	6.06
	F09	0.10	62	5	5.41
	F10	0.05	74	6	5.96
	**Mean**	**0.08**	**60**	**6**	**6.01**
	**S.D.**	**0.02**	**6**	**4**	**0.25**
	**N**	**10**	**10**	**10**	**10**

TBil: total bilirubin, TCho: total cholesterol, TG: triglyceride, TP: total protein.

S.D. indicates standard deviation and N indicates number of animals.

EXCLI Journal 2020;19:187-200 – ISSN 1611-2156
Received: December 12, 2019, accepted: February 05, 2020, published: February 12, 2020

Supplementary Table 3-4: Raw data of clinical chemistry parameters after 2 weeks of recovery period in recovery groups for subchronic toxicity study. The raw data are related to **Table 3**.

Sex: Male

Group / Dose (mg/kg/day)	Animal ID	TBili (mg/dL)	Tcho (mg/dL)	TG (mg/dL)	TP (g/dL)
G1	M11	0.08	41	34	5.54
0 (control)	M12	0.10	46	26	5.58
	M13	0.07	56	17	5.82
	M14	0.06	48	56	5.64
	M15	0.07	61	40	5.83
	M16	0.05	41	43	6.11
	**Mean**	**0.07**	**49**	**36**	**5.75**
	**S.D.**	**0.02**	**8**	**14**	**0.21**
	**N**	**6**	**6**	**6**	**6**
G4	M11	0.08	45	30	5.84
1,000	M12	0.07	44	15	6.07
	M13	0.07	44	21	5.70
	M14	0.05	50	35	5.42
	M15	0.10	34	22	5.87
	M16	0.06	44	29	5.95
	**Mean**	**0.07**	**44**	**25**	**5.81**
	**S.D.**	**0.02**	**5**	**7**	**0.23**
	**N**	**6**	**6**	**6**	**6**

TBil: total bilirubin, TCho: total cholesterol, TG: triglyceride, TP: total protein.

S.D. indicates standard deviation and N indicates number of animals.

EXCLI Journal 2020;19:187-200 – ISSN 1611-2156
Received: December 12, 2019, accepted: February 05, 2020, published: February 12, 2020

Supplementary Table 3-4 (cont.): Raw data of clinical chemistry parameters after 2 weeks of recovery period in recovery groups for subchronic toxicity study. The raw data are related to **Table 3**.

Sex: Female

Group / Dose (mg/kg/day)	Animal ID	TBili (mg/dL)	Tcho (mg/dL)	TG (mg/dL)	TP (g/dL)
G1	F11	0.09	45	11	5.84
0 (control)	F12	0.06	48	10	5.92
	F13	0.07	64	12	5.46
	F14	0.06	62	12	5.81
	F15	0.08	54	15	6.29
	F16	0.07	58	14	6.70
	**Mean**	**0.07**	**55**	**12**	**6.00**
	**S.D.**	**0.01**	**8**	**2**	**0.43**
	**N**	**6**	**6**	**6**	**6**
G4	F11	0.07	47	15	5.80
1,000	F12	0.12	63	10	5.95
	F13	0.08	43	7	6.31
	F14	0.09	44	8	6.08
	F15	0.09	64	13	6.50
	F16	0.09	74	8	6.32
	**Mean**	**0.09**	**56**	**10**	**6.16**
	**S.D.**	**0.02**	**13**	**3**	**0.26**
	**N**	**6**	**6**	**6**	**6**

TBil: total bilirubin, TCho: total cholesterol, TG: triglyceride, TP: total protein.

S.D. indicates standard deviation and N indicates number of animals.

EXCLI Journal 2020;19:187-200 – ISSN 1611-2156
Received: December 12, 2019, accepted: February 05, 2020, published: February 12, 2020

Supplementary Table 4-1: Raw data of absolute and relative organ weights in main groups for sub-chronic toxicity study. The raw data are related to **Table 4**.

Sex: Male

Group / Dose (mg/kg/day)	Animal ID	Body weight (g)	Thymus Absolute (mg)	Thymus Relative (%)	Lung Absolute (g)	Lung Relative (%)	Heart Absolute (g)	Heart Relative (%)
G1	M01	396	761	192.2	1.57	0.396	1.364	0.344
0 (control)	M02	368	621	168.8	1.38	0.375	1.293	0.351
	M03	375	564	150.4	1.31	0.349	1.203	0.321
	M04	414	559	135.0	1.49	0.360	1.239	0.299
	M05	430	798	185.6	1.73	0.402	1.890	0.440
	M06	406	556	136.9	1.37	0.337	1.734	0.427
	M07	381	627	164.6	1.32	0.346	1.247	0.327
	M08	379	501	132.2	1.35	0.356	1.512	0.399
	M09	446	506	113.5	1.47	0.330	1.393	0.312
	M10	321	423	131.8	1.22	0.380	1.376	0.429
	**Mean**	**392**	**592**	**151.1**	**1.42**	**0.363**	**1.425**	**0.365**
	**S.D.**	**35**	**116**	**25.8**	**0.15**	**0.024**	**0.226**	**0.054**
	**N**	**10**	**10**	**10**	**10**	**10**	**10**	**10**
G2	M01	389	691	177.6	1.42	0.365	1.313	0.338
250	M02	373	425	113.9	1.42	0.381	1.378	0.369
	M03	401	614	153.1	1.46	0.364	1.378	0.344
	M04	377	488	129.4	1.46	0.387	1.342	0.356
	M05	371	348	93.8	1.42	0.383	1.207	0.325
	M06	391	691	176.7	1.42	0.363	1.405	0.359
	M07	402	755	187.8	1.50	0.373	1.749	0.435
	M08	388	650	167.5	1.50	0.387	1.394	0.359
	M09	366	588	160.7	1.43	0.391	1.434	0.392
	M10	414	557	134.5	1.44	0.348	1.510	0.365
	**Mean**	**387**	**581**	**149.5**	**1.45**	**0.374**	**1.411**	**0.364**
	**S.D.**	**16**	**128**	**30.7**	**0.03**	**0.014**	**0.143**	**0.031**
	**N**	**10**	**10**	**10**	**10**	**10**	**10**	**10**

S.D. indicates standard deviation and N indicates number of animals.

EXCLI Journal 2020;19:187-200 – ISSN 1611-2156
Received: December 12, 2019, accepted: February 05, 2020, published: February 12, 2020

Supplementary Table 4-1 (cont.): Raw data of absolute and relative organ weights in main groups for subchronic toxicity study. The raw data are related to **Table 4**.

Sex: Male

Group / Dose (mg/kg/day)	Animal ID	Body weight	Thymus		Lung		Heart	
			Absolute	Relative	Absolute	Relative	Absolute	Relative
		(g)	(mg)	(%)	(g)	(%)	(g)	(%)
G3	M01	383	559	146.0	1.53	0.399	1.376	0.359
500	M02	385	414	107.5	1.42	0.369	1.218	0.316
	M03	336	515	153.3	1.33	0.396	1.300	0.387
	M04	377	514	136.3	1.37	0.363	1.147	0.304
	M05	381	594	155.9	1.34	0.352	1.293	0.339
	M06	392	583	148.7	1.43	0.365	1.390	0.355
	M07	402	720	179.1	1.54	0.383	1.246	0.310
	M08	404	657	162.6	1.39	0.344	1.407	0.348
	M09	415	752	181.2	1.58	0.381	1.322	0.319
	M10	386	528	136.8	1.52	0.394	1.351	0.350
	**Mean**	**386**	**584**	**150.7**	**1.45**	**0.375**	**1.305**	**0.339**
	**S.D.**	**21**	**102**	**21.6**	**0.09**	**0.019**	**0.083**	**0.026**
	**N**	**10**	**10**	**10**	**10**	**10**	**10**	**10**
G4	M01	378	448	118.5	1.26	0.333	1.185	0.313
1,000	M02	379	468	123.5	1.31	0.346	1.349	0.356
	M03	410	640	156.1	1.62	0.395	1.330	0.324
	M04	272	307	112.9	1.28	0.471	0.976	0.359
	M05	366	568	155.2	1.39	0.380	1.245	0.340
	M06	331	369	111.5	1.21	0.366	1.138	0.344
	M07	384	430	112.0	1.54	0.401	1.245	0.324
	M08	342	508	148.5	1.40	0.409	1.173	0.343
	M09	393	624	158.8	1.39	0.354	1.332	0.339
	M10	346	426	123.1	1.35	0.390	1.178	0.340
	**Mean**	**360**	**479**	**132.0**	**1.38**	**0.385**	**1.215**	**0.338**
	**S.D.**	**39**	**107**	**20.1**	**0.13**	**0.039**	**0.112**	**0.014**
	**N**	**10**	**10**	**10**	**10**	**10**	**10**	**10**

S.D. indicates standard deviation and N indicates number of animals.

EXCLI Journal 2020;19:187-200 – ISSN 1611-2156
Received: December 12, 2019, accepted: February 05, 2020, published: February 12, 2020

Supplementary Table 4-1 (cont.): Raw data of absolute and relative organ weights in main groups for subchronic toxicity study. The raw data are related to **Table 4**.

Sex: Male

Group / Dose (mg/kg/day)	Animal ID	Body weight	Liver		Kidney		Testis	
			Absolute	Relative	Absolute	Relative	Absolute	Relative
		(g)	(g)	(%)	(g)	(%)	(g)	(%)
G1	M01	396	11.5	2.904	2.93	0.740	3.41	0.861
0 (control)	M02	368	11.1	3.016	2.93	0.796	3.52	0.957
	M03	375	10.7	2.853	3.09	0.824	3.10	0.827
	M04	414	11.6	2.802	2.93	0.708	3.33	0.804
	M05	430	14.1	3.279	3.66	0.851	3.16	0.735
	M06	406	13.6	3.350	3.07	0.756	3.44	0.847
	M07	381	11.4	2.992	3.13	0.822	3.16	0.829
	M08	379	11.2	2.955	2.95	0.778	3.29	0.868
	M09	446	14.0	3.139	3.52	0.789	3.81	0.854
	M10	321	9.7	3.022	2.61	0.813	3.22	1.003
	**Mean**	**392**	**11.9**	**3.031**	**3.08**	**0.788**	**3.34**	**0.859**
	**S.D.**	**35**	**1.5**	**0.177**	**0.31**	**0.043**	**0.21**	**0.075**
	**N**	**10**	**10**	**10**	**10**	**10**	**10**	**10**
G2	M01	389	12.4	3.188	3.26	0.838	2.97	0.763
250	M02	373	10.1	2.708	2.91	0.780	3.57	0.957
	M03	401	12.6	3.142	3.03	0.756	2.87	0.716
	M04	377	11.1	2.944	3.20	0.849	3.39	0.899
	M05	371	11.4	3.073	2.86	0.771	3.17	0.854
	M06	391	11.6	2.967	3.18	0.813	2.80	0.716
	M07	402	11.1	2.761	2.96	0.736	3.31	0.823
	M08	388	12.0	3.093	2.91	0.750	2.88	0.742
	M09	366	10.7	2.923	3.08	0.842	3.60	0.984
	M10	414	12.6	3.043	3.29	0.795	3.06	0.739
	**Mean**	**387**	**11.6**	**2.984**	**3.07**	**0.793**	**3.16**	**0.819**
	**S.D.**	**16**	**0.8**	**0.157**	**0.16**	**0.041**	**0.29**	**0.100**
	**N**	**10**	**10**	**10**	**10**	**10**	**10**	**10**

S.D. indicates standard deviation and N indicates number of animals.

EXCLI Journal 2020;19:187-200 – ISSN 1611-2156
Received: December 12, 2019, accepted: February 05, 2020, published: February 12, 2020

Supplementary Table 4-1 (cont.): Raw data of absolute and relative organ weights in main groups for subchronic toxicity study. The raw data are related to **Table 4**.

Sex: Male

Group / Dose (mg/kg/day)	Animal ID	Body weight	Liver		Kidney		Testis	
			Absolute	Relative	Absolute	Relative	Absolute	Relative
		(g)	(g)	(%)	(g)	(%)	(g)	(%)
G3 500	M01	383	10.8	2.820	3.25	0.844	3.51	0.916
	M02	385	11.5	2.987	2.71	0.807	3.13	0.813
	M03	336	9.5	2.827	2.69	0.714	3.13	0.932
	M04	377	10.1	2.679	3.00	0.787	2.90	0.769
	M05	381	10.7	2.808	3.05	0.778	3.48	0.913
	M06	392	11.5	2.934	3.00	0.746	2.94	0.750
	M07	402	12.3	3.060	3.09	0.765	3.60	0.896
	M08	404	11.5	2.847	3.46	0.834	3.30	0.817
	M09	415	12.2	2.940	3.13	0.811	3.43	0.827
	M10	386	11.1	2.876	1.22	0.380	3.49	0.904
	**Mean**	**386**	**11.1**	**2.878**	**2.86**	**0.747**	**3.29**	**0.854**
	**S.D.**	**21**	**0.9**	**0.107**	**0.62**	**0.135**	**0.25**	**0.066**
	**N**	**10**	**10**	**10**	**10**	**10**	**10**	**10**
G4 1,000	M01	378	10.9	2.884	3.27	0.865	3.11	0.823
	M02	379	11.3	2.982	3.11	0.821	3.24	0.855
	M03	410	12.1	2.951	3.27	0.798	2.84	0.693
	M04	272	7.0	2.574	2.26	0.831	3.84	1.412
	M05	366	9.9	2.705	2.74	0.749	3.40	0.929
	M06	331	8.8	2.659	2.61	0.789	2.91	0.879
	M07	384	11.0	2.865	2.79	0.727	3.47	0.904
	M08	342	9.0	2.632	2.60	0.760	2.93	0.857
	M09	393	10.2	2.595	2.98	0.758	3.34	0.850
	M10	346	9.9	2.861	2.87	0.829	3.38	0.977
	**Mean**	**360**	**10.0**	**2.771**	**2.85**	**0.793**	**3.25**	**0.918**
	**S.D.**	**39**	**1.5**	**0.154**	**0.32**	**0.044**	**0.31**	**0.189**
	**N**	**10**	**10**	**10**	**10**	**10**	**10**	**10**

S.D. indicates standard deviation and N indicates number of animals.

Supplementary Table 4-1 (cont.): Raw data of absolute and relative organ weights in main groups for subchronic toxicity study. The raw data are related to **Table 4**.

Sex: Female

Group / Dose (mg/kg/day)	Animal ID	Body weight	Thymus		Lung		Heart	
			Absolute	Relative	Absolute	Relative	Absolute	Relative
		(g)	(mg)	(%)	(g)	(%)	(g)	(%)
G1	F01	232	412	177.6	1.09	0.470	0.709	0.306
0 (control)	F02	252	517	205.2	1.20	0.476	1.023	0.406
	F03	250	437	174.8	1.15	0.460	0.992	0.397
	F04	231	460	199.1	1.02	0.442	0.784	0.339
	F05	233	391	167.8	1.13	0.485	0.819	0.352
	F06	246	524	213.0	1.15	0.467	0.995	0.404
	F07	234	357	152.6	1.15	0.491	0.858	0.367
	F08	242	533	220.2	1.03	0.426	0.780	0.322
	F09	231	437	189.2	1.08	0.468	0.789	0.342
	F10	219	524	239.3	1.18	0.539	0.861	0.393
	**Mean**	**237**	**459**	**193.9**	**1.12**	**0.472**	**0.861**	**0.363**
	**S.D.**	**10**	**63**	**26.5**	**0.06**	**0.030**	**0.107**	**0.036**
	**N**	**10**	**10**	**10**	**10**	**10**	**10**	**10**
G2	F01	222	359	161.7	1.09	0.491	0.888	0.400
250	F02	234	420	179.5	1.01	0.432	0.863	0.369
	F03	237	674	284.4	1.21	0.511	0.795	0.335
	F04	217	388	178.8	1.08	0.498	0.921	0.424
	F05	270	841	311.5	1.17	0.433	0.942	0.349
	F06	231	557	241.1	1.24	0.537	0.838	0.363
	F07	226	363	160.6	1.08	0.478	0.869	0.385
	F08	217	409	188.5	1.04	0.479	0.927	0.427
	F09	227	553	243.6	1.10	0.485	0.874	0.385
	F10	242	449	185.5	1.19	0.492	0.943	0.390
	**Mean**	**232**	**501**	**213.5**	**1.12**	**0.484**	**0.886**	**0.383**
	**S.D.**	**16**	**157**	**53.3**	**0.08**	**0.032**	**0.048**	**0.030**
	**N**	**10**	**10**	**10**	**10**	**10**	**10**	**10**

S.D. indicates standard deviation and N indicates number of animals.

EXCLI Journal 2020;19:187-200 – ISSN 1611-2156
Received: December 12, 2019, accepted: February 05, 2020, published: February 12, 2020

Supplementary Table 4-1 (cont.): Raw data of absolute and relative organ weights in main groups for subchronic toxicity study. The raw data are related to **Table 4**.

Sex: Female

Group / Dose (mg/kg/day)	Animal ID	Body weight (g)	Thymus Absolute (mg)	Thymus Relative (%)	Lung Absolute (g)	Lung Relative (%)	Heart Absolute (g)	Heart Relative (%)
G3	F01	242	534	220.7	1.14	0.471	0.899	0.371
500	F02	250	575	230.0	1.26	0.504	0.846	0.338
	F03	206	371	180.1	0.95	0.461	0.830	0.403
	F04	242	514	212.4	1.22	0.504	0.950	0.393
	F05	237	476	200.8	1.17	0.494	0.997	0.421
	F06	210	630	300.0	1.03	0.490	0.898	0.428
	F07	242	455	188.0	1.24	0.512	0.968	0.400
	F08	229	412	179.9	1.10	0.480	0.795	0.347
	F09	223	413	185.2	1.06	0.475	0.827	0.371
	F10	238	493	207.1	1.19	0.500	0.933	0.392
	**Mean**	**232**	**487**	**210.4**	**1.14**	**0.489**	**0.894**	**0.386**
	**S.D.**	**15**	**79**	**35.9**	**0.10**	**0.017**	**0.068**	**0.030**
	**N**	**10**	**10**	**10**	**10**	**10**	**10**	**10**
G4	F01	214	244	114.0	0.96	0.449	0.937	0.438
1,000	F02	218	443	203.2	1.07	0.491	0.822	0.377
	F03	217	316	145.6	1.15	0.530	0.921	0.424
	F04	218	326	149.5	1.18	0.541	0.904	0.415
	F05	220	371	168.6	1.09	0.495	0.772	0.351
	F06	254	398	156.7	1.21	0.476	0.932	0.367
	F07	238	350	147.1	1.11	0.466	0.963	0.405
	F08	220	454	206.4	1.20	0.545	0.976	0.444
	F09	230	484	210.4	1.25	0.543	1.066	0.463
	F10	231	379	164.1	1.07	0.463	0.966	0.418
	**Mean**	**226**	**377**	**166.6**	**1.13**	**0.500**	**0.926**	**0.410**
	**S.D.**	**12**	**72**	**31.3**	**0.09**	**0.037**	**0.082**	**0.036**
	**N**	**10**	**10**	**10**	**10**	**10**	**10**	**10**

S.D. indicates standard deviation and N indicates number of animals.

EXCLI Journal 2020;19:187-200 – ISSN 1611-2156
Received: December 12, 2019, accepted: February 05, 2020, published: February 12, 2020

Supplementary Table 4-1 (cont.): Raw data of absolute and relative organ weights in main groups for subchronic toxicity study. The raw data are related to **Table 4**.

Sex: Female

Group / Dose (mg/kg/day)	Animal ID	Body weight (g)	Liver Absolute (g)	Liver Relative (%)	Kidney Absolute (g)	Kidney Relative (%)
G1	F01	232	5.7	2.457	1.53	0.659
0 (control)	F02	252	7.2	2.857	2.20	0.873
	F03	250	7.7	3.080	2.17	0.868
	F04	231	6.4	2.771	1.81	0.784
	F05	233	6.4	2.747	1.96	0.841
	F06	246	7.3	2.967	2.15	0.874
	F07	234	6.3	2.692	1.70	0.726
	F08	242	7.3	3.017	1.86	0.769
	F09	231	6.0	2.597	1.67	0.723
	F10	219	6.4	2.922	1.67	0.763
	**Mean**	**237**	**6.7**	**2.811**	**1.87**	**0.788**
	**S.D.**	**10**	**0.7**	**0.196**	**0.24**	**0.074**
	**N**	**10**	**10**	**10**	**10**	**10**
G2	F01	222	6.2	2.793	1.70	0.766
250	F02	234	6.8	2.906	1.83	0.782
	F03	237	7.3	3.080	1.74	0.734
	F04	217	6.7	3.088	1.99	0.917
	F05	270	8.3	3.074	2.11	0.781
	F06	231	7.2	3.117	1.79	0.775
	F07	226	6.3	2.788	1.94	0.858
	F08	217	6.7	3.088	1.86	0.857
	F09	227	7.0	3.084	1.76	0.775
	F10	242	7.8	3.223	1.92	0.793
	**Mean**	**232**	**7.0**	**3.024**	**1.86**	**0.804**
	**S.D.**	**16**	**0.6**	**0.145**	**0.13**	**0.055**
	**N**	**10**	**10**	**10**	**10**	**10**

S.D. indicates standard deviation and N indicates number of animals.

EXCLI Journal 2020;19:187-200 – ISSN 1611-2156
Received: December 12, 2019, accepted: February 05, 2020, published: February 12, 2020

Supplementary Table 4-1 (cont.): Raw data of absolute and relative organ weights in main groups for subchronic toxicity study. The raw data are related to Table 4.

Sex: Female

Group / Dose (mg/kg/day)	Animal ID	Body weight (g)	Liver Absolute (g)	Liver Relative (%)	Kidney Absolute (g)	Kidney Relative (%)
G3	F01	242	7.9	3.264	1.99	0.822
500	F02	250	7.1	2.840	1.98	0.792
	F03	206	5.7	2.767	1.71	0.830
	F04	242	7.0	2.893	1.72	0.711
	F05	237	7.1	2.996	1.90	0.802
	F06	210	6.3	3.000	1.85	0.881
	F07	242	7.3	3.017	1.98	0.818
	F08	229	6.7	2.926	1.85	0.808
	F09	223	6.5	2.915	1.94	0.870
	F10	238	6.9	2.899	1.72	0.723
	**Mean**	**232**	**6.9**	**2.952**	**1.86**	**0.806**
	**S.D.**	**15**	**0.6**	**0.134**	**0.11**	**0.055**
	**N**	**10**	**10**	**10**	**10**	**10**
G4	F01	214	5.6	2.617	1.67	0.780
1,000	F02	218	6.6	3.028	1.84	0.844
	F03	217	6.7	3.088	1.70	0.783
	F04	218	6.2	2.844	1.66	0.761
	F05	220	6.3	2.864	2.00	0.909
	F06	254	7.4	2.913	1.85	0.728
	F07	238	7.2	3.025	1.73	0.727
	F08	220	6.9	3.136	1.67	0.759
	F09	230	7.2	3.130	1.76	0.765
	F10	231	7.0	3.030	1.89	0.818
	**Mean**	**226**	**6.7**	**2.968**	**1.78**	**0.787**
	**S.D.**	**12**	**0.6**	**0.161**	**0.11**	**0.056**
	**N**	**10**	**10**	**10**	**10**	**10**

S.D. indicates standard deviation and N indicates number of animals.

EXCLI Journal 2020;19:187-200 – ISSN 1611-2156
Received: December 12, 2019, accepted: February 05, 2020, published: February 12, 2020

Supplementary Table 4-2: Raw data of absolute and relative organ weights in recovery groups for subchronic toxicity study. The raw data are related to **Table 4**.

Sex: Male

Group / Dose (mg/kg/day)	Animal ID	Body weight	Thymus		Lung		Heart	
			Absolute	Relative	Absolute	Relative	Absolute	Relative
		(g)	(mg)	(%)	(g)	(%)	(g)	(%)
G1	M11	418	417	99.8	1.33	0.318	1.606	0.384
0 (control)	M12	422	455	107.8	1.38	0.327	1.531	0.363
	M13	389	302	77.6	1.40	0.360	1.316	0.338
	M14	524	402	76.7	1.66	0.317	1.692	0.323
	M15	466	395	84.8	1.55	0.333	1.543	0.331
	M16	426	353	82.9	1.36	0.319	1.268	0.298
	**Mean**	**441**	**387**	**88.3**	**1.45**	**0.329**	**1.493**	**0.340**
	**S.D.**	**48**	**53**	**12.7**	**0.13**	**0.016**	**0.166**	**0.030**
	**N**	**6**	**6**	**6**	**6**	**6**	**6**	**6**
G4	M11	403	456	113.2	1.50	0.372	1.539	0.382
1,000	M12	407	439	107.9	1.63	0.400	1.303	0.320
	M13	397	674	169.8	1.43	0.360	1.295	0.326
	M14	453	663	146.4	1.54	0.340	1.364	0.301
	M15	400	396	99.0	1.42	0.355	1.651	0.413
	M16	430	533	124.0	1.55	0.360	1.516	0.353
	**Mean**	**415**	**527**	**126.7**	**1.51**	**0.365**	**1.445**	**0.349**
	**S.D.**	**22**	**118**	**26.7**	**0.08**	**0.020**	**0.145**	**0.042**
	**N**	**6**	**6**	**6**	**6**	**6**	**6**	**6**

S.D. indicates standard deviation and N indicates number of animals.

EXCLI Journal 2020;19:187-200 – ISSN 1611-2156
Received: December 12, 2019, accepted: February 05, 2020, published: February 12, 2020

Supplementary Table 4-2 (cont.): Raw data of absolute and relative organ weights in recovery groups for subchronic toxicity study. The raw data are related to **Table 4**.

Sex: Male

Group / Dose (mg/kg/day)	Animal ID	Body weight	Liver		Kidney		Testis	
			Absolute	Relative	Absolute	Relative	Absolute	Relative
		(g)	(g)	(%)	(g)	(%)	(g)	(%)
G1	M11	418	10.8	2.584	2.95	0.706	2.84	0.679
0 (control)	M12	422	11.2	2.654	2.96	0.701	3.50	0.829
	M13	389	9.8	2.519	2.68	0.689	3.35	0.861
	M14	524	16.2	3.092	4.04	0.771	3.92	0.748
	M15	466	14.1	3.026	3.50	0.751	3.48	0.747
	M16	426	12.6	2.958	2.53	0.594	3.13	0.735
	**Mean**	**441**	**12.5**	**2.806**	**3.11**	**0.702**	**3.37**	**0.767**
	**S.D.**	**48**	**2.4**	**0.248**	**0.56**	**0.062**	**0.37**	**0.067**
	**N**	**6**	**6**	**6**	**6**	**6**	**6**	**6**
G4	M11	403	11.1	2.754	3.14	0.779	3.49	0.866
1,000	M12	407	11.1	2.727	3.16	0.776	3.49	0.857
	M13	397	11.0	2.771	3.20	0.806	3.46	0.872
	M14	453	12.9	2.848	3.79	0.837	4.12	0.909
	M15	400	12.0	3.000	3.46	0.865	3.51	0.878
	M16	430	11.6	2.698	3.24	0.753	3.75	0.872
	**Mean**	**415**	**11.6**	**2.800**	**3.33**	**0.803**	**3.64**	**0.876**
	**S.D.**	**22**	**0.7**	**0.110**	**0.25**	**0.042**	**0.26**	**0.018**
	**N**	**6**	**6**	**6**	**6**	**6**	**6**	**6**

S.D. indicates standard deviation and N indicates number of animals.

EXCLI Journal 2020;19:187-200 – ISSN 1611-2156
Received: December 12, 2019, accepted: February 05, 2020, published: February 12, 2020

Supplementary Table 4-2 (cont.): Raw data of absolute and relative organ weights in recovery groups for subchronic toxicity study. The raw data are related to **Table 4**.

Sex: Female

Group / Dose (mg/kg/day)	Animal ID	Body weight	Thymus		Lung		Heart	
			Absolute	Relative	Absolute	Relative	Absolute	Relative
		(g)	(mg)	(%)	(g)	(%)	(g)	(%)
G1	F11	212	331	156.1	0.95	0.448	0.782	0.369
0 (control)	F12	253	426	168.4	1.17	0.462	0.976	0.386
	F13	280	337	120.4	1.23	0.439	0.978	0.349
	F14	253	237	93.7	1.13	0.447	0.936	0.370
	F15	245	415	169.4	1.11	0.453	0.921	0.376
	F16	250	365	146.0	1.09	0.436	0.798	0.319
	**Mean**	**249**	**352**	**142.3**	**1.11**	**0.448**	**0.899**	**0.362**
	**S.D.**	**22**	**69**	**29.9**	**0.09**	**0.009**	**0.087**	**0.024**
	**N**	**6**	**6**	**6**	**6**	**6**	**6**	**6**
G4	F11	269	417	155.0	1.28	0.476	0.853	0.317
1,000	F12	264	439	166.3	1.22	0.462	0.865	0.328
	F13	229	286	124.9	1.13	0.493	0.806	0.352
	F14	272	439	161.4	1.24	0.456	1.074	0.395
	F15	250	352	140.8	1.09	0.436	0.915	0.366
	F16	274	563	205.5	1.23	0.449	0.935	0.341
	**Mean**	**260**	**416**	**159.0**	**1.20**	**0.462**	**0.908**	**0.350**
	**S.D.**	**17**	**93**	**27.3**	**0.07**	**0.020**	**0.093**	**0.028**
	**N**	**6**	**6**	**6**	**6**	**6**	**6**	**6**

S.D. indicates standard deviation and N indicates number of animals.

EXCLI Journal 2020;19:187-200 – ISSN 1611-2156
Received: December 12, 2019, accepted: February 05, 2020, published: February 12, 2020

Supplementary Table 4-2 (cont.): Raw data of absolute and relative organ weights in recovery groups for subchronic toxicity study. The raw data are related to **Table 4**.

Sex: Female

Group / Dose (mg/kg/day)	Animal ID	Body weight	Liver		Kidney	
			Absolute	Relative	Absolute	Relative
		(g)	(g)	(%)	(g)	(%)
G1	F11	212	6.0	2.830	1.62	0.764
0 (control)	F12	253	7.1	2.806	1.79	0.708
	F13	280	7.3	2.607	1.93	0.689
	F14	253	7.2	2.846	1.82	0.719
	F15	245	6.8	2.776	1.90	0.776
	F16	250	7.5	3.000	1.74	0.696
	**Mean**	**249**	**7.0**	**2.811**	**1.80**	**0.725**
	**S.D.**	**22**	**0.5**	**0.127**	**0.11**	**0.036**
	**N**	**6**	**6**	**6**	**6**	**6**
G4	F11	269	7.3	2.714	2.37	0.881
1,000	F12	264	7.3	2.765	2.01	0.761
	F13	229	6.8	2.969	1.86	0.812
	F14	272	8.3	3.051	1.86	0.684
	F15	250	6.7	2.680	1.72	0.688
	F16	274	7.4	2.701	2.10	0.766
	**Mean**	**260**	**7.3**	**2.813**	**1.99**	**0.765**
	**S.D.**	**17**	**0.6**	**0.157**	**0.23**	**0.075**
	**N**	**6**	**6**	**6**	**6**	**6**

S.D. indicates standard deviation and N indicates number of animals.
